# Prenatal exposure to maternal asthma and asthma medication and neurodevelopmental outcomes: a population cohort study of 179,024 children

**DOI:** 10.1186/s12916-026-04699-x

**Published:** 2026-02-20

**Authors:** Lama A. Shakhshir, Sarjit Singh, Jill P. Pell, Scott M. Nelson, Daniel F. Mackay, Claire E. Hastie, Logesh R. Sivakumar, Michael Fleming

**Affiliations:** 1 School of Health and Wellbeing, University of Glasgow, Clarice Pears Building, 90 Byres Road, Glasgow, G12 8TB UK; 2 School of Medicine, Dentistry and Nursing, University of Glasgow, Glasgow, UK

**Keywords:** Anti-asthmatic medication, Asthma, Attention deficit disorder with hyperactivity, Autism spectrum disorder, Learning disorders, Special educational need, In-utero exposure

## Abstract

**Background:**

Asthma exacerbations during pregnancy can adversely affect maternal and foetal health. However, prenatal exposure to asthma medication may itself impact child outcomes. This study aims to determine whether prenatal exposure to asthma medication was associated with a range of neurodevelopmental outcomes.

**Methods:**

A birth cohort was constructed using linked health and education records of children liveborn in Wales from 2009 to 2016, with up to 12 years follow-up. Generalized estimating equations with a binomial distribution and logit link function were employed to evaluate special educational need (SEN) and its causes, including autism spectrum disorder (ASD), communication problems, behavioural, emotional, and social difficulties, learning difficulties, physical and medical difficulties, and sensory impairment. Cox proportional hazard models were used to investigate attention deficit hyperactivity disorder (ADHD).

**Results:**

Of the 179,024 offspring, 11,991 (6.7%) had mothers with treated asthma, 4927 (2.8%) had mothers with untreated asthma, and 5265 (2.9%) had mothers who took asthma medication without a record of asthma. SEN was recorded in 50,955 (28.5%) children. Risk of overall SEN and ADHD was higher following prenatal exposure to short-acting beta-agonists (SABA) and inhaled corticosteroids (ICS) but also higher in the offspring of mothers with untreated asthma (SEN aOR 1.14, 95% CI 1.07–1.23; ADHD aHR 1.70, 95% CI 1.42–2.05). ASD was associated with treated, but not untreated, asthma and the association with prenatal medication was specific to SABA as mono- or polytherapy (aOR 1.20, 95% CI 1.05–1.37). However, a direct comparison of treated with untreated asthma was not statistically significant.

**Conclusions:**

The non-specific associations with overall SEN and ADHD are likely to reflect their associations with asthma and confounding by indication/severity. The associations with ASD may reflect a treatment effect of SABA or confounding by severity. Further studies are needed to confirm or refute the former, to support evidence-based asthma management during pregnancy.

**Supplementary Information:**

The online version contains supplementary material available at 10.1186/s12916-026-04699-x.

## Background

Asthma is a chronic condition with a prevalence of 8–13% among pregnant women worldwide [1]. Since uncontrolled asthma risks both maternal and foetal health [2, 3], clinical guidelines recommend continuation of asthma medication through pregnancy [4]. Substantial evidence supports the safety of β_2_ adrenergic receptor agonists (B2AA) and inhaled corticosteroids (ICS) in terms of maternal and perinatal outcomes including preeclampsia, preterm birth, low birthweight, and birth defects [5–7]. Fewer studies have investigated longer-term neurodevelopmental outcomes, and their results are inconsistent [8–12]. One reported an association between prenatal exposure to B2AA and ADHD [10]. Of four studies investigating ASD, two reported an association [8, 11] and two did not [9, 13]. No associations have been reported with cerebral palsy, communication, gross and fine motor function, problem-solving, and personal-social skills [12, 14].

B2AA is the usual first-line treatment, but asthma exacerbations increase during pregnancy [15], often requiring adjuvant therapies. Previous studies largely ignored other classes of asthma medication [8, 10, 11, 16]. Other limitations included inconsistencies regarding whether and how studies differentiated between effects of the condition—asthma—and its treatment, and use of asthma medications for other indications, such as use of B2AA and corticosteroids for preterm labour [17–20].

Using a large, unselected birth cohort we investigated associations between treated and untreated maternal asthma and a range of neurodevelopmental outcomes, including ASD, ADHD, and learning difficulties, and determined whether associations were specific to one or more classes of asthma medication.

## Methods

A birth cohort was constructed using the Secure Anonymised Information Linkage (SAIL) databank which covers 86% of the Welsh population (> 3 million). Six datasets were linked: the Welsh Longitudinal General Practice Dataset (WLGP), the National Community Child Health Database (NCCHD), the Annual District Birth Extracts (ADBE), the Pupil Level Annual School Census (PLASC) data within the Education Wales database (EDUW), the Welsh Demographic Service Dataset (WDSD), and the Maternity Indicators Dataset (MIDS). We included livebirths from 2009 to 2016 and excluded mothers not registered with a SAIL practitioner for two full years before delivery and offspring with missing education data (e.g. educated outside of Wales).

The exposures were maternal asthma (treated and untreated) and maternal asthma medication (overall and by class). Asthma was defined as at least one eligible Read code (Additional file 1: Supplementary Table 1) recorded within 2 years prior to delivery. Asthma medication was defined as at least one eligible Read code (Additional file 1: Supplementary Table 2) documented from conception to delivery. Date of conception was derived by subtracting gestational age at delivery plus 2 weeks from date of delivery. Asthma medications were categorised into 21 groups based on the National Institute for Health and Care Excellence (NICE) treatment guidelines (Additional file 1: Supplementary Table 3).

The primary outcome was a record of special educational need (SEN). Secondary outcomes were types of SEN: ASD, sensory impairment, communication problems, physical and medical difficulties, learning difficulties, and behavioural, emotional, and social difficulties. SEN data were updated annually, and a child could have more than one type of SEN recorded. ADHD was ascertained from SEN attributed to ADHD, a primary care diagnosis of ADHD (Additional file 1: Supplementary Table 4), or one or more prescriptions for ADHD medication (Additional file 1: Supplementary Table 5) at any point from birth. Maximum follow-up was 12 years from birth.

Potential confounders were sociodemographic factors (child’s sex, age, ethnicity, and socioeconomic status), maternal factors (maternal age, and smoking status during pregnancy), and pregnancy factors (parity, and multiple births (yes/no)) (Additional file 1: Supplementary Fig. 1). Socioeconomic status was measured as quintiles of the Welsh Index of Multiple Deprivation (WIMD) 2014 [21] based on residential postcode at birth. Potential mediators included birthweight centile, mode of delivery, gestational age at delivery, and 5-min Apgar score (Additional file 1: Supplementary Fig. 1).

For the primary analyses, the comparison group was offspring of women with no record of asthma nor asthma medication. We compared with the comparison group, offspring of: women with any indication of asthma (record of asthma or asthma medication); women with treated asthma (record of asthma and record of asthma medication) and untreated asthma (record of asthma; no record of asthma medication); women prescribed any asthma medication (with or without a record of asthma); and women prescribed each of the 21 medication classes with sufficient statistical power. The latter analyses were conducted both including and excluding mothers receiving other asthma medications; polytherapy and monotherapy respectively.

Group characteristics were summarized using frequencies with percentages and means with standard deviations and compared using χ^2^ tests, χ^2^ tests for trend, and *t*-tests for categorical, ordinal, and continuous variables respectively. For ADHD, we ran Cox proportional hazard models. For all other outcomes, we applied logistic regression models using generalized estimating equations with a binomial distribution and logit link function to account for correlations between repeated SEN records across multiple school years [22], and selected the structure with the lowest QIC [23]. The first model was unadjusted, the second adjusted for sociodemographic factors, the third also adjusted for maternal and pregnancy factors, and the final model included potential mediators. For the two variables with the highest missing data (maternal smoking status and mode of delivery), missing values were treated as dummy variables. For the secondary analyses, we repeated the models for treated asthma using untreated asthma as the referent group.

The study was approved by the Information Governance Review Panel and was covered by a Data Sharing Agreement between the SAIL Databank and the University of Glasgow. All statistical analyses were performed using Stata/SE v18.0.

## Results

Between 2009 and 2016, 200,152 children were born in Wales to mothers registered with SAIL general practitioners over the previous 2 years. Of these, 21,128 were excluded: 1312 were stillborn and 19,816 could not be linked to Welsh educational records. Of the 179,024 eligible children (173,849 singletons), 22,183 (12.4%) had mothers with a record of asthma or asthma medication: 11,991 (6.7%) had mothers with treated asthma, 4927 (2.8%) had mothers with untreated asthma, and 5265 (2.9%) had mothers who received asthma medication without a record of asthma.

The baseline characteristics of mothers with any evidence of asthma (record of asthma or asthma medication) are contained in Additional file 1: Supplementary Table 6, and the characteristics of all of the asthma categories are contained in Table 1. Women with any evidence of asthma were more likely to smoke, deliver preterm, and have offspring with smaller birthweight centiles (Table 1, Additional file 1: Supplementary Table 6). Compared to mothers with no evidence of asthma, those with untreated asthma were younger and more likely to be nulliparous, whilst those with treated asthma, or receiving asthma medication, were more likely to be multiparous, live in deprived areas and undergo Caesarean section (Table 1). Of the 17,256 mothers prescribed asthma medication, 16,304 (94.5%) took SABAs, 6102 (35.4%) ICS, and 4936 (28.6%) combination LABA and ICS. Less common medications included LABA (378, 2.2%) and leukotriene antagonists (LTRA) (375, 2.2%). Other asthma medications were each used by less than 1% of mothers. Overall, 50,955 children (28.5%) had a record of SEN. Of these, 28,088 (55.1%) had learning difficulties, 20,291 (39.8%) communication problems, 12,115 (23.8%) behavioural, emotional, and social difficulties, 3668 (7.2%) ASD, 3344 (6.6%) physical and medical difficulties and 1977 (3.9%) sensory impairment. ADHD or its treatment was documented for 3287 (1.8%) children.

**Table 1:** Characteristics and outcomes of cohort participants by asthma category

	**No maternal asthma**		**Untreated asthma**		***P*-value**	**Treated asthma**		***P*-value**	**Asthma medication without asthma diagnosis**		***P*-value**
	***N*** = 156,841		***N*** = 4927			***N*** = 11,991			***N*** = 5265		
	Mean (SD)		Mean (SD)			Mean (SD)			Mean (SD)		
	*N*	**%**	*N*	**%**		*N*	%		*N*	**%**	
**Child’s age (years)**	5.68 (1.11)		5.68 (1.12)		0.571	5.67 (1.11)		0.873	5.66 (1.11)		0.348
**Child's sex**											
Female	76,868	49.5	2380	48.3	0.330	5796	48.3	0.155	2602	49.4	0.558
Male	79,973	50.5	2547	51.7		6195	51.7		2663	50.6	
**Child’s ethnicity**											
White	146,972	95.4	4747	96.4	< 0.001	11,410	95.2	< 0.001	4937	93.8	0.053
Asian/Asian British	3290	1.2	26	0.5		149	1.2		87	1.7	
Black/Black British	752	0.3	8	0.2		21	0.2		22	0.4	
Dual heritage	4730	2.6	123	2.5		350	2.9		184	3.5	
Other	992	0.4	20	0.4		53	0.4		34	0.6	
Missing	105	-	3	-		8	-		1	-	
**Maternal age (years)**											
< 25	42,871	27.6	1683	34.2	< 0.001	3266	27.2	0.585	1482	28.1	0.708
25–29	46,079	29.8	1555	31.6		3526	29.4		1541	29.3	
30–34	42,033	26.5	1103	22.4		3176	26.5		1324	25.1	
≥ 35	25,841	16.1	586	11.9		2021	16.9		918	17.4	
Missing	17	-	0	-		2	-		0	-	
**Maternal smoking status**											
Non-smoker	65,564	41.8	2055	61.1	< 0.001	5370	57.3	< 0.001	1952	53.9	< 0.001
Ex smoker	2315	1.5	110	3.3		435	4.6		82	2.3	
Quit smoking during pregnancy	5112	3.3	236	7.0		747	8.0		204	5.6	
Current smoker	25,296	16.1	964	28.6		2812	30.0		1385	38.2	
Missing	58,554	37.3	1562	-		2627	-		1642	-	
**Birthweight centile**											
1–3	5017	3.0	179	3.6	0.029	435	3.7	< 0.001	202	3.9	0.006
4–10	11,382	7.1	382	7.8		990	8.3		395	7.6	
11–20	15,841	10.2	495	10.1		1260	10.6		583	11.2	
21–80	93,273	60.2	2931	59.7		7029	59.1		3062	58.6	
81–90	15,247	9.7	446	9.1		1105	9.3		484	9.3	
91–97	10,664	6.9	354	7.2		728	6.1		333	6.4	
98–100	4633	2.9	121	2.5		356	3.0		164	3.1	
Missing	784	-	19	-		88	-		42	-	
**Gestational age (weeks)**											
< 28	401	0.1	17	0.3	0.002	20	0.2	0.002	8	0.2	0.012
28–32	1785	1.0	59	1.2		127	1.1		57	1.1	
33–36	8693	5.6	333	6.8		679	5.7		325	6.2	
37	9130	6.1	327	6.7		805	6.7		333	6.4	
38	18,454	12.2	579	11.8		1533	12.9		657	12.6	
39	35,407	22.9	1042	21.2		2693	22.6		1172	22.4	
40	43,477	27.7	1366	27.8		3137	26.3		1421	27.2	
41	32,063	20.6	965	19.6		2406	20.2		1036	19.8	
≥ 42	6821	3.8	224	4.6		527	4.4		220	4.2	
Missing	610	-	15	-		64	-		36	-	
**Mode of delivery**											
Spontaneous vaginal	66,866	42.6	2030	59.3	0.057	4873	59.1	< 0.001	2293	61.7	0.007
Assisted	12,106	7.7	408	11.9		942	11.4		364	9.8	
Breech	430	0.3	20	0.6		28	0.3		19	0.5	
Elective CS	12,430	7.9	411	12.0		1073	13.0		459	12.3	
Emergency CS	16,502	10.5	553	16.2		1330	16.1		583	15.7	
Missing	48,507	30.9	1505	-		3745	-		1547	-	
**Parity**											
0	65,647	45.5	2227	50.8	< 0.001	5150	48.6	0.673	2029	43.4	< 0.001
1	44,977	33.5	1346	30.7		3241	30.6		1449	31.0	
≥ 2	27,732	21.0	808	18.4		2210	20.8		1194	25.6	
Missing	18,485	-	546	-		1390	-		593	-	
**5-min Apgar score**											
0–3	278	0.2	6	0.1	0.209	27	0.2	0.101	7	0.1	0.966
4–6	1495	1.1	41	0.9		127	1.1		55	1.1	
7–10	147,600	98.7	4647	99.0		11,275	98.7		4993	98.8	
Missing	7468	-	233	-		562	-		210	-	
**WIMD quintile**											
1 (most deprived)	41,213	24.6	1272	25.8	0.805	3303	27.6	< 0.001	1554	29.5	< 0.001
2	34,833	23.1	1107	22.5		2668	22.3		1187	22.5	
3	30,872	20.9	994	20.2		2369	19.8		1025	19.5	
4	24,657	16.9	802	16.3		1810	15.1		795	15.1	
5 (least deprived)	25,223	14.5	750	15.2		1838	15.3		703	13.4	
Missing	43	-	2	-		3	-		1	-	
**Number of births**											
1 (singletons)	152,283	97.3	4785	97.1	0.921	11,658	97.2	0.417	5123	97.3	0.374
≥ 2 (multiples)	4558	2.7	142	2.9		333	2.8		142	2.7	
**Special educational need**	43,754	27.9	1556	31.6	< 0.001	3931	32.8	< 0.001	1714	32.6	< 0.001
ASD	3103	2.0	128	2.6	0.002	296	2.5	< 0.001	141	2.7	0.001
Communication problems	17,485	11.1	626	12.7	< 0.001	1521	12.7	< 0.001	659	12.5	0.002
Behavioural, emotional, and social difficulties	10,216	6.5	435	8.8	< 0.001	1004	8.4	< 0.001	460	8.7	< 0.001
Learning difficulties	24,135	15.4	830	16.8	0.005	2190	18.3	< 0.001	933	17.7	< 0.001
Physical and medical difficulties	2823	1.8	114	2.3	0.008	297	2.5	< 0.001	110	2.1	0.121
Sensory impairment	1676	1.1	79	1.6	< 0.001	171	1.4	< 0.001	51	1	0.487
**ADHD**	2688	1.7	149	3.0	< 0.001	328	2.7	< 0.001	122	2.3	0.001

*SD* standard deviation, *N* number, *CS* caesarean section, *WIMD* Welsh Index of Multiple Deprivation, *ASD* autism spectrum disorder, *ADHD* attention deficit hyperactivity disorder

All *p*-values related to comparisons with the no maternal asthma group

**Fig. 1:**
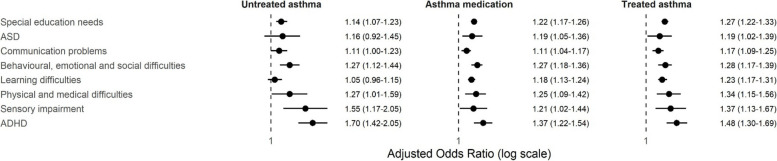
Associations between untreated and treated maternal asthma and asthma medications and childhood outcomes, referent to women with no record of asthma diagnosis or medication. ADHD attention deficit hyperactivity disorder; ASD autism spectrum disorder; HR hazard ratio; OR odds ratio. All models adjusted for socioeconomic, maternal, and pregnancy confounders

In comparison to women with no evidence of asthma, maternal asthma (record of asthma or asthma medication) was associated with overall SEN, all types of SEN and ADHD, and remained so after adjusting for potential sociodemographic, maternal and pregnancy factors (Table 2). Further adjustment for potential mediators produced minimal attenuation (Table 2). All-cause SEN, SEN attributed to physical and medical, and behavioural, emotional, and social difficulties, and ADHD were associated with treated asthma and asthma medication, but also untreated asthma (Fig. 1 and Additional file 1: Supplementary Table 7). In contrast, SEN attributed to ASD, learning difficulties and communication problems was associated specifically with treated asthma and asthma medication, and not with untreated asthma (Fig. 1 and Additional file 1: Supplementary Table 7).

**Table 2:** Associations between maternal asthma^*^ and childhood outcomes

	**Unadjusted**		**Adjusted for sociodemographic confounders**		**Adjusted for sociodemographic, maternal and pregnancy confounders**		**Adjusted for confounders and mediators**	
	*N* = 179,024		*N* = 178,975		*N* = 157,959		*N* = 151,262	
	OR (95% CI)	*P*-value	OR (95% CI)	*P*-value	OR (95% CI)	*P*-value	OR (95% CI)	*P*-value
Special educational need	1.25 (1.21–1.29)	< 0.001	1.24 (1.20–1.28)	< 0.001	1.20 (1.16–1.24)	< 0.001	1.20 (1.16–1.25)	< 0.001
ASD	1.23 (1.10–1.36)	< 0.001	1.21 (1.09–1.35)	< 0.001	1.18 (1.06–1.33)	0.004	1.18 (1.05–1.33)	0.004
Communication problems	1.16 (1.10–1.21)	< 0.001	1.14 (1.09–1.20)	< 0.001	1.11 (1.05–1.17)	< 0.001	1.11 (1.05–1.17)	< 0.001
Behavioural, emotional, and social difficulties	1.36 (1.28–1.44)	< 0.001	1.33 (1.26–1.41)	< 0.001	1.27 (1.19–1.35)	< 0.001	1.27 (1.19–1.36)	< 0.001
Learning difficulties	1.21 (1.16–1.26)	< 0.001	1.19 (1.15–1.24)	< 0.001	1.15 (1.10–1.21)	< 0.001	1.16 (1.10–1.21)	< 0.001
Physical and medical difficulties	1.23 (1.10–1.37)	< 0.001	1.22 (1.10–1.36)	< 0.001	1.25 (1.11–1.41)	< 0.001	1.30 (1.15–1.47)	< 0.001
Sensory impairment	1.24 (1.08–1.43)	0.003	1.23 (1.07–1.42)	0.004	1.29 (1.11–1.50)	0.001	1.30 (1.11–1.52)	0.001
	*N* = 160,261		*N* = 160,234		*N* = 141,940		*N* = 135,933	
	HR (95% CI)	*P*-value	HR (95% CI)	*P*-value	HR (95% CI)	*P*-value	HR (95% CI)	*P*-value
ADHD	1.55 (1.41–1.70)	< 0.001	1.51 (1.38–1.66)	< 0.001	1.45 (1.31–1.61)	< 0.001	1.44 (1.29–1.60)	< 0.001

^*^Record of asthma diagnosis and/or record of asthma medication

*N* number, *CI* confidence interval, *ASD* autism spectrum disorder, *ADHD* attention deficit hyperactivity disorder, *HR* hazard ratio, *OR* odds ratio

All-cause SEN, SEN attributed to learning difficulties, and ADHD were associated with SABA, either used as monotherapy or with another medication, and with ICS combined with either LABA or another medication (Table 3). Any exposure to SABA was associated with ASD (aOR 1.20, 95% CI 1.05–1.37, *p* = 0.006). SABA monotherapy had the same effect size but did not reach statistical significance (aOR 1.20, 95% CI 0.98–1.46, *p* = 0.72). ASD was not associated with ICS monotherapy or with ICS and LABA combination therapy, but was associated with ICS used in conjunction with SABA (aOR 1.34, 95% CI 1.07–1.67, *p* = 0.01) (Additional file 1: Supplementary Table 8).

**Table 3:** Associations between maternal asthma medication class and childhood outcomes

	**SABA with/without other class**		**SABA without other class**	
	*N* = 157,725		*N* = 144,178	
	OR (95% CI)	*P*-value	OR (95% CI)	*P*-value
Special educational need	1.23 (1.18–1.28)	< 0.001	1.13 (1.07–1.20)	< 0.001
ASD	1.20 (1.05–1.37)	0.006	1.20 (0.98–1.46)	0.072
Communication problems	1.12 (1.05–1.18)	< 0.001	0.99 (0.90–1.09)	0.828
Behavioural, emotional, and social difficulties	1.29 (1.20–1.39)	< 0.001	1.28 (1.15–1.43)	< 0.001
Learning difficulties	1.19 (1.14–1.26)	< 0.001	1.11 (1.03–1.20)	0.007
Physical and medical difficulties	1.23 (1.08–1.41)	0.003	1.04 (0.83–1.30)	0.751
Sensory impairment	1.18 (0.99–1.41)	0.072	0.93 (0.69–1.25)	0.635
	*N* = 137,271		*N* = 129,572	
	HR (95% CI)	*P*-value	HR (95% CI)	*P*-value
ADHD	1.39 (1.23–1.56)	< 0.001	1.23 (1.02–1.48)	0.029
	**ICS with/without other class**		**ICS without other class**	
	*N* = 143,688		*N* = 138,640	
	OR (95% CI)	*P*-value	OR (95% CI)	*P*-value
Special educational need	1.28 (1.20–1.36)	< 0.001	0.95 (0.74–1.24)	0.728
ASD	1.27 (1.04–1.56)	0.017	1.55 (0.76–3.16)	0.228
Communication problems	1.18 (1.07–1.29)	< 0.001	0.91 (0.62–1.34)	0.639
Behavioural, emotional, and social difficulties	1.24 (1.10–1.39)	< 0.001	0.62 (0.34–1.12)	0.115
Learning difficulties	1.25 (1.16–1.35)	< 0.001	0.98 (0.70–1.37)	0.900
Physical and medical difficulties	1.28 (1.04–1.57)	0.020	1.49 (0.70–3.18)	0.296
Sensory impairment	1.39 (1.06–1.82)	0.017	2.61 (1.12–6.10)	0.026
	*N* = 129,131		*N* = 124,611	
	HR (95% CI)	*P*-value	HR (95% CI)	*P*-value
ADHD	1.51 (1.26–1.79)	< 0.001	0.78 (0.29–2.09)	0.625
	**ICS plus LABA**^**a**^** with/without other class**		**ICS plus LABA**^**a**^** without other class**	
	*N* = 142,687		*N* = 138,763	
	OR (95% CI)	*P*-value	OR (95% CI)	*P*-value
Special educational need	1.28 (1.20–1.37)	< 0.001	1.07 (0.86–1.35)	0.536
ASD	1.05 (0.83–1.33)	0.670	0.78 (0.30–2.00)	0.607
Communication problems	1.23 (1.11–1.36)	< 0.001	1.04 (0.74–1.46)	0.843
Behavioural, emotional, and social difficulties	1.29 (1.13–1.47)	< 0.001	1.08 (0.69–1.69)	0.740
Learning difficulties	1.22 (1.12–1.33)	< 0.001	0.90 (0.66–1.22)	0.508
Physical and medical difficulties	1.48 (1.19–1.84)	< 0.001	1.65 (0.89–3.07)	0.112
Sensory impairment	1.51 (1.13–2.02)	0.006	1.44 (0.63–3.32)	0.388
	*N* = 128,288		*N* = 124,724	
	HR (95% CI)	*P*-value	HR (95% CI)	*P*-value
ADHD	1.41 (1.15–1.73)	< 0.001	1.25 (0.62–2.50)	0.531
	**LABA with/without other class**		**LABA without other class**^**b**^	
	*N* = 138,641		*N* = 138,314	
	OR (95% CI)	*P*-value	OR (95% CI)	*P*-value
Special educational need	1.48 (1.18–1.86)	< 0.001	-	-
ASD	0.58 (0.22–1.48)	0.254	-	-
Communication problems	1.15 (0.80–1.63)	0.453	-	-
Behavioural, emotional, and social difficulties	1.44 (0.95–2.20)	0.089	-	-
Learning difficulties	1.32 (1.00–1.75)	0.050	-	-
Physical and medical difficulties	1.33 (0.68–2.62)	0.405	-	-
Sensory impairment	2.34 (0.96–5.67)	0.060	-	-
	*N* = 124,604		*N* = 124,317	
	HR (95% CI)	*P*-value	HR (95% CI)	*P*-value
ADHD	2.28 (1.35–3.86)	0.002	-	-
	**LTRA with/without other class**		**LTRA without other class**^**b**^	
	*N* = 138,636		*N* = 138,321	
	OR (95% CI)	*P*-value	OR (95% CI)	*P*-value
Special educational need	1.59 (1.24–2.02)	< 0.001	-	-
ASD	1.24 (0.63–2.43)	0.538	-	-
Communication problems	1.01 (0.71–1.44)	0.951	-	-
Behavioural, emotional, and social difficulties	2.15 (1.44–3.22)	< 0.001	-	-
Learning difficulties	1.37 (1.01–1.85)	0.043	-	-
Physical and medical difficulties	3.36 (1.84–6.13)	< 0.001	-	-
Sensory impairment	1.54 (0.44–5.38)	0.503	-	-
	*N* = 124,611		*N* = 124,324	
	HR (95% CI)	*P*-value	HR (95% CI)	*P*-value
ADHD	2.52 (1.39–4.56)	0.002	-	-

*N* number, *CI* confidence interval, *ASD* autism spectrum disorder, *ADHD* attention deficit hyperactivity disorder, *HR* hazard ratio

*OR* odds ratio, *SABA* short-acting beta-agonists, *ICS* inhaled corticosteroids, *LABA* long-acting beta-agonists, *LTRA* leukotrienes antagonists

^a^ “ICS plus LABA” represents the fixed-dose combination inhaler that contains both active ingredients

^b^ Number of mothers on LABA as a monotherapy and LTRA as a monotherapy was less than 10

In comparison to women with untreated asthma, treated asthma was associated with all-cause SEN and SEN due to learning difficulties, after adjustment for confounders (Table 4). There was no significant association with ASD and ADHD.

**Table 4:** Associations between treated asthma and childhood outcomes, referent to untreated asthma

	**Unadjusted**		**Adjusted for sociodemographic confounders**		**Adjusted for sociodemographic, maternal and pregnancy confounders**		**Adjusted for confounders and mediators**	
	*N* = 16,918		*N* = 16,913		*N* = 14,976		*N* = 14,327	
	OR (95% CI)	*P*-value	OR (95% CI)	*P*-value	OR (95% CI)	*P*-value	OR (95% CI)	*P*-value
Special educational need	1.03 (0.96–1.11)	0.447	1.04 (0.96–1.12)	0.303	1.12 (1.03–1.22)	0.006	1.13 (1.04–1.23)	0.005
ASD	0.97 (0.76–1.24)	0.837	0.98 (0.77–1.25)	0.881	1.06 (0.81–1.39)	0.675	1.02 (0.78–1.34)	0.880
Communication problems	0.98 (0.87–1.10)	0.727	0.98 (0.88–1.10)	0.794	1.06 (0.94–1.20)	0.342	1.09 (0.96–1.23)	0.206
Learning difficulties	1.08 (0.98–1.19)	0.101	1.10 (1.00–1.21)	0.061	1.17 (1.06–1.31)	0.003	1.18 (1.06–1.32)	0.003
	*N* = 16,918		*N* = 16,902		*N* = 14,965		*N* = 14,316	
	OR (95% CI)	*P*-value	OR (95% CI)	*P*-value	OR (95% CI)	*P*-value	OR (95% CI)	*P*-value
Behavioural, emotional, and social difficulties	0.94 (0.82–1.08)	0.366	0.96 (0.83–1.10)	0.517	1.01 (0.87–1.17)	0.873	1.04 (0.89–1.20)	0.650
	*N* = 16,918		*N* = 16,874		*N* = 14,940		*N* = 14,291	
	OR (95% CI)	*P*-value	OR (95% CI)	*P*-value	OR (95% CI)	*P*-value	OR (95% CI)	*P*-value
Physical and medical difficulties	1.07 (0.84–1.38)	0.576	1.08 (0.84–1.38)	0.564	1.03 (0.79–1.35)	0.803	1.07 (0.81–1.39)	0.643
	*N* = 16,918		*N* = 16,818		*N* = 14,878		*N* = 14,232	
	OR (95% CI)	*P*-value	OR (95% CI)	*P*-value	OR (95% CI)	*P*-value	OR (95% CI)	*P*-value
Sensory impairment	0.87 (0.64–1.20)	0.395	0.88 (0.64–1.20)	0.407	0.88 (0.63–1.23)	0.457	0.85 (0.61–1.19)	0.354
	*N* = 15,137		*N* = 15,133		*N* = 13,420		*N* = 12,847	
	HR (95% CI)	*P*-value	HR (95% CI)	*P*-value	HR (95% CI)	*P*-value	HR (95% CI)	*P*-value
ADHD	0.86 (0.70–1.06)	0.154	0.87 (0.71–1.06)	0.170	0.89 (0.72–1.12)	0.324	0.93 (0.74–1.17)	0.540

*N* number, *CI* confidence interval, *ASD* autism spectrum disorder, *ADHD* attention deficit hyperactivity disorder, *HR* hazard ratio, *OR* odds ratio

Sample sizes for the outcomes “behavioural, emotional and social difficulties”, “physical and medical difficulties” and “sensory impairment” differ from those of other outcomes because certain categories of the variable “child’s ethnicity” were automatically omitted due to lack of variation in these outcomes

## Discussion

In this large, unselected birth cohort, ASD was associated with prenatal exposure to asthma medication and, specifically, exposure to SABA but not untreated maternal asthma. The association with SABA was independent of sociodemographic, maternal, and pregnancy factors and did not appear to be mediated by factors such as preterm delivery, intrauterine growth restriction, or mode of delivery. Whilst these findings could be explained by a causal relationship between prenatal SABA exposure and ASD, a direct comparison of treated and untreated asthma was not statistically significant; therefore, confounding by severity could not be ruled out. The findings for ASD contrasted with those for all-cause SEN, learning difficulties, and ADHD, which had less-specific associations with asthma medication, including both SABA and ICS, and were also associated with untreated maternal asthma, although all-cause SEN and learning difficulties had significantly stronger associations with treated than untreated asthma. These non-specific findings suggest that the associations with these outcomes were likely explained by confounding by indication and possibly severity.

Two studies have previously reported associations between prenatal exposure to B2AA and ASD, and suggested that B2AA exposure may affect neurodevelopment independently of a maternal asthma diagnosis [8, 11]. However, both investigated B2AA use as a whole; neither investigated exposure to SABAs specifically. A causal association between prenatal exposure to B2AA generally, and SABAs specifically, and neurodevelopmental outcomes is biologically plausible. A potential pharmacological effect on foetal brain development could possibly occur through mechanisms such as altered neurotransmitter signalling or inflammatory pathways [24]. In animal models, high doses of B2AAs have been shown to cause lasting dysregulation of beta-2 adrenergic receptor (B2AR) signalling [25–27]. This dysregulation is associated with changes in brain microarchitecture, including the cerebellum, hippocampus, and cortex, functional differences in cell signalling across developmental stages, behavioural changes, and neuroinflammation in juvenile rats.

Our finding that ADHD was associated with untreated as well as treated asthma is consistent with previous findings reported by Liang et al., who demonstrated a similar risk of ADHD among the offspring of women using SABA during pregnancy and the offspring of those using SABA in the 2 years pre-conception only [10]. Liu et al. also reported increased risk of ADHD in the offspring of fathers with asthma, noting that poorly controlled parental asthma was more strongly associated with offspring ADHD risk than well-controlled asthma, regardless of whether the asthma was maternal or paternal [28]. These findings suggest that the associations between maternal asthma and ADHD may be due to shared environmental factors and/or genetic predisposition, rather than maternal health or maternal medication.

Our finding of a significant association between prenatal exposure to ICS monotherapy and sensory impairment might be counterintuitive since ICS is used as a maintenance therapy and, therefore, patients on ICS might be presumed to have well-controlled asthma. Whilst it may be a spurious finding, there is also existing evidence of plausibility since in their study, Uberos et al. found that prenatal corticosteroids may be associated with neurosensory alterations in full-term infants [29]. Therefore, this finding should be considered as hypothesis-generating and merits further investigation.

Our study was a large-scale, unselected birth cohort study. Secondary analysis of routinely collected data helped to obviate selection, recall, and reporting biases and provided a long follow-up period and data on a wide range of outcomes. We were able to differentiate between pregnant women treated for asthma and those with untreated asthma, and our study had sufficient power to perform subgroup analyses for the most commonly used classes of asthma medication: SABAs, ICS, and LABAs combined with ICS. Previous studies have often lacked the statistical power to distinguish between different subtypes of B2AA, such as SABAs and LABAs.

However, our study also had limitations. One key limitation was the inability to conduct subgroup analyses for all 21 classes of asthma medications due to small sample sizes of some classes. Additionally, the medication data pertained to prescriptions issued, not actual medication intake, meaning we cannot be certain that mothers took the medications as prescribed, or that any non-adherence with medication occurred randomly. We identified a subset of women who were prescribed asthma medication without a recorded diagnosis of asthma. While some of these women may have been taking the medication for other indications, under-recording of asthma diagnoses or diagnoses made before the 2-year look-back period could also explain use of these medications. Nonetheless, our findings were consistent when the analyses were repeated excluding women taking asthma medications without a recorded diagnosis of asthma. In our cohort, most women prescribed SABAs used salbutamol rather than terbutaline (8.69% versus 0.51%), with salbutamol typically used as a reliever therapy. Therefore, confounding by severity is possible in that SABA use could reflect more severe or poorly controlled asthma. Although some asthmatic patients may be prescribed single-agent maintenance and reliever therapy (MART), the number of patients on MART in our study was low, likely due to it not being included in the British Thoracic Society asthma guidelines until 2019 [30]. In observational studies, confounding by indication is always possible but we attempted to mitigate this risk by investigating women with both treated asthma and untreated asthma, by comparing the former to both untreated asthma and no asthma, and by exploring whether associations with medications were specific to medication class.

It is possible that the non-significant association between untreated asthma and ASD reflected insufficient statistical power. Our study sample provided 90% power to detect an OR of 1.34 or greater. Our effect estimates for ASD ranged from 1.16 to 1.19 and had wider confidence intervals than some of the other outcomes. Our findings are, nonetheless, consistent with previous studies that also reported non-significant associations between untreated asthma and ASD[31, 32] but the inclusion of our findings in a future meta-analysis would be prudent to corroborate the observed lack of association with untreated asthma with greater statistical power. Our direct comparison between treated and untreated asthma did not produce statistically significant results. The wide confidence intervals again suggest that this comparison should be tested in future, larger studies or meta-analyses with greater statistical power.

The SAIL databank currently covers 83% of Welsh family practitioners. It is unlikely that the practices that submit data to SAIL differed systematically from those who did not, in terms of underlying prevalence of maternal asthma or SEN, which minimizes the risk of selection bias. Of the children born during the study period, less than 1% could not be linked to their family practitioner records, typically due to moving to a Welsh family practitioner who did not participate in SAIL or moving outside of Wales. We adjusted for a wide range of sociodemographic, maternal, and pregnancy confounders; however, we did not have access to some potential confounding factors, such as maternal adiposity, and residual confounding is always possible in any observational study. Birth records could not be linked to education records for those children who had not yet started school and those who were educated outside of Wales (e.g. the family lived close to the border and the child attended school in England or the family relocated after birth). If there were differences between this group and our cohort in terms of general characteristics, such as the socioeconomic status, prevalence estimates might not be generalisable but effect estimates, such as odds ratios, should still be generalisable. Finally, changes in asthma diagnosis or management over time could introduce temporal bias.

## Conclusions

While the continued use of asthma medications is crucial to prevent exacerbations and protect maternal and foetal health, it is also important to understand and consider the potential risks associated with specific medications to make informed management decisions. In contrast to ADHD and other neurodevelopmental outcomes, ASD was associated with prenatal exposure to SABA medication, and to ICS, but not with other asthma medications or untreated asthma. Whilst this would be consistent with prenatal exposure to SABA being causally associated with ASD, the lack of a statistically significant result in the direct comparison between untreated and treated asthma means that confounding by severity cannot be refuted as an alternative explanation. Therefore, further large-scale studies and/or meta-analyses, with large statistical power, are essential to explore these alternative explanations.

## Supplementary Information

Additional file 1. Supplementary Fig. 1 and Supplementary Tables 1–8. Supplementary Fig. 1: Directed acyclic graph (DAG) of confounder and mediator variables in the relationship between maternal asthma and child outcomes. Supplementary Table 1: Read codes for asthma diagnosis. Supplementary Table 2: Read codes for asthma medication. Supplementary Table 3: Medication groups. Supplementary Table 4: Read codes for ADHD diagnosis. Supplementary Table 5: Read codes for ADHD medications. Supplementary Table 6: Characteristics and outcomes of cohort participants by presence or absence of maternal asthma. Supplementary Table 7: Associations between treated and untreated maternal asthma and childhood outcomes. Supplementary Table 8: Associations between different combinations of classes of asthma medications and childhood outcomes.

## Data Availability

The authors applied for permission to access, link, and analyse these data within the SAIL databank which is a trusted research environment. To do this, the researchers were required to undertake mandatory training in data protection, IT security and information governance. Therefore, the datasets generated and analysed during the study are not publicly available. Interested researchers may apply directly to SAIL for data access to health and education data by emailing [SAILDatabank@swansea.ac.uk](mailto:SAILDatabank@swansea.ac.uk).

## Abbreviations

ADBE: Annual District Birth Extracts
ADHD: Attention deficit hyperactivity disorder
ASD: Autism spectrum disorder
B2AA: β2 Adrenergic receptor agonists
CS: Caesarean section
EDUW: Education Wales database
ICS: Inhaled corticosteroids
LABA: Long-acting beta-agonist
LTRA: Leukotrienes antagonists
MART: Maintenance and reliever therapy
MIDS: Maternity Indicators Dataset
NCCHD: National Community Child Health Database
NICE: National Institute for Health and Care Excellence
PLASC: Pupil Level Annual School Census
SABA: Short-acting beta-agonist
SAIL: Secure Anonymised Information Linkage
SEN: Special educational need
WDSD: Welsh Demographic Service Dataset
WIMD: Welsh Index of Multiple Deprivation
WLGP: Welsh Longitudinal General Practice Dataset
