# Supplementary material for: Prenatal exposure to maternal asthma and asthma medication and neurodevelopmental outcomes: a population cohort study of 179,024 children
Source: BMC Med. 2026 Feb 20;24:178. doi: 10.1186/s12916-026-04699-x (PMC13032591; doi:10.1186/s12916-026-04699-x)
Supplement: Supplementary file 1 — Additional file 1. Supplementary Fig. 1 and Supplementary Tables 1–8. Supplementary Fig. 1: Directed acyclic graph (DAG) of confounder and mediator variables in the relationship between maternal asthma and child outcomes. Supplementary Table 1: Read codes for asthma diagnosis. Supplementary Table 2: Read codes for asthma medication. Supplementary Table 3: Medication groups. Supplementary Table 4: Read codes for ADHD diagnosis. Supplementary Table 5: Read codes for ADHD medications. Supplementary Table 6: Characteristics and outcomes of cohort participants by presence or absence of maternal asthma. Supplementary Table 7: Associations between treated and untreated maternal asthma and childhood outcomes. Supplementary Table 8: Associations between different combinations of classes of asthma medications and childhood outcomes. [file 12916_2026_4699_MOESM1_ESM.docx]

**Additional file 1**

**Supplementary Figure 1. Directed acyclic graph (DAG) of confounder and mediator variables in the relationship between maternal asthma and child outcomes.**

**Supplementary Table 1. Read codes for asthma diagnosis.**

**Supplementary Table 2. Read codes for asthma medication.**

**Supplementary Table 3. Medication groups.**

**Supplementary Table 4. Read codes for ADHD diagnosis.**

**Supplementary Table 5. Read codes for ADHD medications.**

**Supplementary Table 6. Characteristics and outcomes of cohort participants by presence or absence of maternal asthma**

**Supplementary Table 7. Associations between treated and untreated maternal asthma and childhood outcomes**

**Supplementary Table 8. Associations between different combinations of classes of asthma medications and childhood outcomes**

**Supplementary Figure 1.** **Directed acyclic graph (DAG) of confounder and mediator variables in the relationship between maternal asthma and child outcomes.** ^[[1]](#footnote-1)^


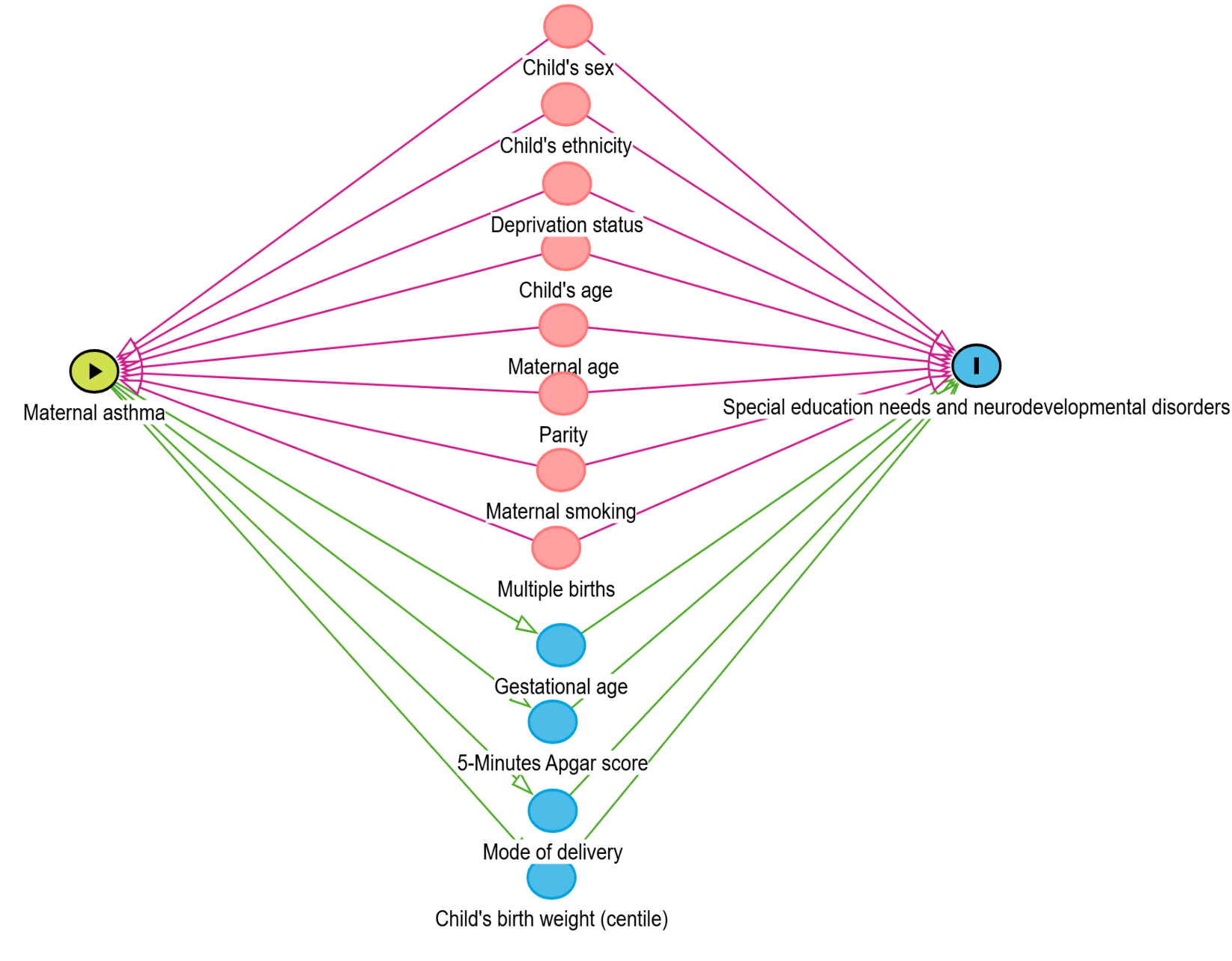


**Supplementary Table 1. Read codes for asthma diagnosis**

| **#** | **Read codes for asthma as a diagnosis** | **Primary description** | **Secondary descriptions in case of term codes** ^a^ |
| --- | --- | --- | --- |
| 1 | 14B4. | H/O: asthma |  |
| 2 | 173A. | Exercise-induced asthma |  |
| 3 | 173c. | Occupational asthma |  |
| 4 | 173d. | Work aggravated asthma |  |
| 5 | 178.. | Asthma trigger |  |
| 6 | 1780. | Aspirin-induced asthma |  |
| 7 | 1781. | Asthma trigger - pollen |  |
| 8 | 1782. | Asthma trigger - tobacco smoke |  |
| 9 | 1783. | Asthma trigger - warm air |  |
| 10 | 1784. | Asthma trigger - emotion |  |
| 11 | 1785. | Asthma trigger - damp |  |
| 12 | 1786. | Asthma trigger: animals |  |
| 13 | 1787. | Asthma trigger - seasonal |  |
| 14 | 1788. | Asthma trigger - cold air |  |
| 15 | 1789. | Asthma trigger - respiratory infection |  |
| 16 | 178A. | Asthma trigger - airborne dust |  |
| 17 | 178B. | Asthma trigger - exercise |  |
| 18 | 1O2.. | Asthma confirmed |  |
| 19 | 38DL. | Asthma control test score |  |
| 20 | 38DT. | Asthma Control Questionnaire score |  |
| 21 | 38DV. | Mini AQLQ (Asthma Quality of Life Questionnaire) score |  |
| 22 | 38QM. | Childhood Asthma Control Test score |  |
| 23 | 661M1 | Asthma self-management plan agreed |  |
| 24 | 661N1 | Asthma self-management plan review |  |
| 25 | 663d. | Emergency asthma admission since last encounter |  |
| 26 | 663e. | Asthma restricts exercise |  |
| 27 | 663e0 | Asthma sometimes restricts exercise |  |
| 28 | 663e1 | Asthma severely restricts exercise |  |
| 29 | 663f. | Asthma never restricts exercise |  |
| 30 | 663h. | Asthma - currently dormant |  |
| 31 | 663j. | Asthma - currently active |  |
| 32 | 663m. | Emergency asthma patient visit since last encounter |  |
| 33 | 663N. | Asthma disturbing sleep |  |
| **#** | **Read codes for asthma as a diagnosis** | **Primary description** | **Secondary descriptions in case of Term codes** ^a^ |
| 34 | 663n. | Asthma treatment compliance satisfactory |  |
| 35 | 663N0 | Asthma causing night waking |  |
| 36 | 663N1 | Asthma disturbs sleep weekly |  |
| 37 | 663N2 | Asthma disturbs sleep frequently |  |
| 38 | 663O. | Asthma not disturbing sleep |  |
| 39 | 663O0 | Asthma never disturbs sleep |  |
| 40 | 663P. | Asthma limiting activities |  |
| 41 | 663p. | Asthma treatment compliance unsatisfactory |  |
| 42 | 663P0 | Asthma limits activities 1 to 2 times per month |  |
| 43 | 663P1 | Asthma limits activities 1 to 2 times per week |  |
| 44 | 663P2 | Asthma limits activities most days |  |
| 45 | 663Q. | Asthma not limiting activities |  |
| 46 | 663q. | Asthma daytime symptoms |  |
| 47 | 663r. | Asthma causes night symptoms 1 to 2 times per month |  |
| 48 | 663s. | Asthma never causes daytime symptoms |  |
| 49 | 663t. | Asthma causes daytime symptoms 1 to 2 times per month |  |
| 50 | 663U. | Asthma management |  |
| 51 | 663u. | Asthma causes daytime symptoms 1 to 2 times per week |  |
| 52 | 663V. | Asthma severity |  |
| 53 | 663v. | Asthma causes daytime symptoms most days |  |
| 54 | 663V0 | Occasional asthma |  |
| 55 | 663V1 | Mild asthma |  |
| 56 | 663V2 | Moderate asthma |  |
| 57 | 663V3 | Severe asthma |  |
| 58 | 663W. | Asthma prophylactic medication used |  |
| 59 | 663w. | Asthma limits walking up hills or stairs |  |
| 60 | 663x. | Asthma limits walking on the flat |  |
| 61 | 663y. | Number of asthma exacerbations in past year |  |
| 62 | 66Y5. | Change in asthma management plan |  |
| 63 | 66Y9. | Step up change in asthma management plan |  |
| **#** | **Read codes for asthma as a diagnosis** | **Primary description** | **Secondary descriptions in case of Term codes** ^a^ |
| 64 | 66YA. | Step down change in asthma management plan |  |
| 65 | 66YC. | Absent from work or school due to asthma |  |
| 66 | 66YE. | Asthma monitoring due |  |
| 67 | 66YJ. | Asthma annual review |  |
| 68 | 66YK. | Asthma follow-up |  |
| 69 | 66YP. | Asthma night-time symptoms |  |
| 70 | 66Yp. | Asthma review using Royal College of Physicians three questions |  |
| 71 | 66YQ. | Asthma monitoring by nurse |  |
| 72 | 66Yq. | Asthma causes night time symptoms 1 to 2 times per week |  |
| 73 | 66YR. | Asthma monitoring by doctor |  |
| 74 | 66Yr. | Asthma causes symptoms most nights |  |
| 75 | 66Ys. | Asthma never causes night symptoms |  |
| 76 | 66Yu. | Number of days absent from school due to asthma in past 6 months |  |
| 77 | 66Yz0 | Asthma management plan declined |  |
| 78 | 66Yz5 | Telehealth asthma monitoring |  |
| 79 | 679J. | Health education - asthma |  |
| 80 | 679J0 | Health education - asthma self management |  |
| 81 | 679J1 | Health education - structured asthma discussion |  |
| 82 | 679J2 | Health education - structured patient focused asthma discussion |  |
| 83 | 8791. | Further asthma - drug prevention |  |
| 84 | 8793. | Asthma control step 0 |  |
| 85 | 8794. | Asthma control step 1 |  |
| 86 | 8795. | Asthma control step 2 |  |
| 87 | 8796. | Asthma control step 3 |  |
| 88 | 8797. | Asthma control step 4 |  |
| 89 | 8798. | Asthma control step 5 |  |
| 90 | 8B3j. | Asthma medication review |  |
| 91 | 8CE2. | Asthma leaflet given |  |
| 92 | 8CMA0 | Patient has a written asthma personal action plan |  |
| 93 | 8CR0. | Asthma clinical management plan |  |
| **#** | **Read codes for asthma as a diagnosis** | **Primary description** | **Secondary descriptions in case of Term codes** ^a^ |
| 94 | 8H2P. | Emergency hospital admission for asthma |  |
| 95 | 8HTT. | Referral to asthma clinic |  |
| 96 | 9hA.. | Exception reporting - asthma quality indicators |  |
| 97 | 9hA1. | Excepted from asthma quality indicators - patient unsuitable |  |
| 98 | 9hA2. | Excepted from asthma quality indicators - informed dissent |  |
| 99 | 9N4Q. | Did not attend asthma clinic |  |
| 100 | 9NI8. | Asthma outreach clinic |  |
| 101 | 9NNX. | Under care of asthma specialist nurse |  |
| 102 | 9OJ.. | Asthma monitoring administration | Asthma clinic administration |
| 103 | 9OJ1. | Attends asthma monitoring |  |
| 104 | 9OJ2. | Asthma monitoring refused |  |
| 105 | 9OJ3. | Asthma monitor offer default |  |
| 106 | 9OJ4. | Asthma monitoring call first letter |  |
| 107 | 9OJ5. | Asthma monitoring call second letter |  |
| 108 | 9OJ6. | Asthma monitoring call third letter |  |
| 109 | 9OJ7. | Asthma monitoring call verbal invite |  |
| 110 | 9OJ8. | Asthma monitoring call telephone invite |  |
| 111 | 9OJ9. | Asthma monitoring deleted |  |
| 112 | 9OJA. | Asthma monitoring check done | Asthma monitored |
| 113 | 9OJB. | Asthma monitoring invitation SMS (short message service) text message |  |
| 114 | 9OJB0 | Asthma monitoring SMS (short message service) text message first invitation |  |
| 115 | 9OJB1 | Asthma monitoring SMS (short message service) text message second invitation |  |
| 116 | 9OJB2 | Asthma monitoring SMS (short message service) text message third invitation |  |
| 117 | 9OJC. | Asthma monitoring invitation email |  |
| 118 | 9OJZ. | Asthma monitoring admin.NOS |  |
| 119 | 9Q21. | Patient in asthma study |  |
| 120 | H3120 | Chronic asthmatic bronchitis | Chronic wheezy bronchitis |
| 121 | H33.. | Asthma | Bronchial asthma |
| 122 | H330. | Extrinsic (atopic) asthma | Pollen asthma |
|  |  |  | Allergic asthma |
|  |  |  | Childhood asthma |
| **#** | **Read codes for asthma as a diagnosis** | **Primary description** | **Secondary descriptions in case of Term codes** ^a^ |
|  |  |  | Hay fever with asthma |
| 123 | H3300 | Extrinsic asthma without status asthmaticus | Hay fever with asthma |
| 124 | H3301 | Extrinsic asthma with status asthmaticus | Extrinsic asthma with asthma attack |
| 125 | H330z | Extrinsic asthma |  |
| 126 | H331. | Intrinsic asthma | Late onset asthma |
| 127 | H3310 | Intrinsic asthma without status asthmaticus |  |
| 128 | H3311 | Instrinsic asthma with status asthmaticus | Instrinsic asthma with asthma attack |
| 129 | H331z | Intrinsic asthma NOS |  |
| 130 | H332. | Mixed asthma |  |
| 131 | H333. | Acute exacerbation of asthma |  |
| 132 | H334. | Brittle asthma |  |
| 133 | H335. | Chronic asthma with fixed airflow obstruction |  |
| 134 | H33z. | Asthma unspecified | Hyperreactive airway disease |
| 135 | H33z0 | Status asthmaticus NOS | Severe asthma attack |
| 136 | H33z1 | Asthma attack | Asthma attack NOS |
| 137 | H33z2 | Late-onset asthma |  |
| 138 | H33zz | Asthma NOS | Allergic bronchitis NEC |
|  |  |  | Exercise induced asthma |
|  |  |  | Allergic asthma NEC |
| 139 | H35y6 | Sequoiosis (red-cedar asthma) |  |
| 140 | H35y7 | Wood asthma |  |
| 141 | H3B.. | Asthma-chronic obstructive pulmonary disease overlap syndrome |  |
| 142 | H47y0 | Detergent asthma |  |
| 143 | 9N1d. | Seen in asthma clinic |  |
| 144 | 9N1d0 | Seen in school asthma clinic |  |
| 145 | 663J | Airways obstruction reversible |  |

Term codes are Read codes that have more than one description.

**Supplementary Table 2. Read codes for asthma medication**

| **#** | **Read codes for asthma medications** | **Description** |
| --- | --- | --- |
| 1 | c1… | SELECTIVE BETA-ADRENOCEPTOR STIMULANT |
| 2 | c11.. | SALBUTAMOL [ORAL PREPARATIONS] |
| 3 | c111. | *ASMAVEN 2mg tablets |
| 4 | c112. | *ASMAVEN 4mg tablets |
| 5 | c113. | *COBUTOLIN 2mg tablets |
| 6 | c114. | *COBUTOLIN 4mg tablets |
| 7 | c115. | *SALBULIN 2mg tablets |
| 8 | c116. | *SALBULIN 4mg tablets |
| 9 | c117. | *SALBULIN 2mg/2mL liquid |
| 10 | c118. | *VENTOLIN 2mg tablets |
| 11 | c119. | *VENTOLIN 4mg tablets |
| 12 | c11a. | *VENTOLIN 8mg m/r tablets |
| 13 | c11A. | *VENTOLIN CR 4mg m/r tablets |
| 14 | c11b. | VENTOLIN 2mg/5mL syrup |
| 15 | c11B. | *SALBUTAMOL 4mg m/r tablets |
| 16 | c11c. | *VOLMAX 4mg m/r tablets |
| 17 | c11C. | *VENTOLIN CR 8mg m/r tablets |
| 18 | c11d. | *VOLMAX 8mg m/r tablets |
| 19 | c11D. | SALAPIN 2mg/5mL sugar free syrup |
| 20 | c11e. | *SALBUVENT 2mg tablets |
| 21 | c11f. | *SALBUVENT 4mg tablets |
| 22 | c11g. | *SALBUVENT 2mg/5mL syrup |
| 23 | c11h. | SALBUVENT 2mg/5mL syrup 2litre |
| 24 | c11i. | *VENTOLIN CR 4mg m/r tablets |
| 25 | c11j. | *SALBUTAMOL 4mg m/r tablets |
| 26 | c11k. | *VENTOLIN CR 8mg m/r tablets |
| 27 | c11m. | LIBETIST 2mg/5mL sugar free syrup |
| 28 | c11n. | *SALBUTAMOL 4mg m/r capsules |
| 29 | c11o. | *SALBUTAMOL 8mg m/r capsules |
| 30 | c11p. | *VENTMAX SR 4mg m/r capsules |
| 31 | c11q. | *VENTMAX SR 8mg m/r capsules |
| 32 | c11v. | SALBUTAMOL 4mg tablets |
| 33 | c11w. | *SALBUTAMOL 2mg/2mL liquid |
| 34 | c11x. | SALBUTAMOL 2mg tablets |
| 35 | c11y. | *SALBUTAMOL 8mg m/r tablets |
| **#** | **Read codes for asthma medications** | **Description** |
| 36 | c11z. | SALBUTAMOL 2mg/5mL sugar free syrup |
| 37 | c12.. | SALBUTAMOL [PARENTERAL PREPARATIONS] |
| 38 | c121. | VENTOLIN 250micrograms/5mL injection |
| 39 | c122. | VENTOLIN 500microgram/1mL injection |
| 40 | c123. | VENTOLIN 5mg/5mL intravenous infusion |
| 41 | c124. | SALBUVENT 250microgram/5mL injection |
| 42 | c125. | SALBUVENT 500micrograms/1mL injection |
| 43 | c126. | SALBUVENT 5mg/5mL intravenous infusion |
| 44 | c12w. | *SALBUTAMOL 5mg/50mL injection |
| 45 | c12x. | SALBUTAMOL 250micrograms/5mL injection |
| 46 | c12y. | SALBUTAMOL 500microgram/1mL injection |
| 47 | c12z. | SALBUTAMOL 5mg/5mL intravenous infusion |
| 48 | c13.. | SALBUTAMOL [INHALATION PREPRATIONS] |
| 49 | c131. | *ASMAVEN 100micrograms inhaler |
| 50 | c132. | COBUTOLIN 100microgram inhaler |
| 51 | c133. | SALBULIN 100micrograms inhaler |
| 52 | c134. | VENTOLIN 100micrograms inhaler |
| 53 | c135. | VENTOLIN 2.5mg/2.5mL Nebules |
| 54 | c136. | VENTOLIN 200micrograms rotacaps |
| 55 | c137. | VENTOLIN 400micrograms rotacaps |
| 56 | c138. | ROTAHALER DEVICE |
| 57 | c139. | VENTOLIN 100mg/20mL respirator solution |
| 58 | c13a. | AEROLIN-400 100microgram inhaler |
| 59 | c13A. | STERI-NEB SALAMOL 2.5mg nebulisation units |
| 60 | c13b. | *ROTAHALER DEVICE |
| 61 | c13B. | STERI-NEB SALAMOL 5mg nebulisation units |
| 62 | c13c. | AEROLIN AUTO 100microgram inhaler |
| 63 | c13C. | SALBUTAMOL 200micrograms disks+disk inhaler |
| 64 | c13d. | VENTODISKS 200micrograms diskhaler 14x8 |
| 65 | c13D. | SALBUTAMOL 400micrograms disks+disk inhaler |
| 66 | c13e. | VENTODISKS 400micrograms diskhaler 14x8 |
| 67 | c13E. | SALBUTAMOL 200micrograms disk refill |
| 68 | c13f. | VENTODISKS 200micrograms disk refill 14x8 |
| 69 | c13F. | SALBUTAMOL 400micrograms disk refill |
| 70 | c13g. | VENTODISKS 400micrograms disk refill 14x8 |
| 71 | c13G. | SALBUTAMOL 100micrograms breath-act aerosol inhaler |
| 72 | c13h. | SALBUVENT 100micrograms inhaler |
| **#** | **Read codes for asthma medications** | **Description** |
| 73 | c13H. | *SALAMOL 100micrograms inhaler |
| 74 | c13i. | SALBUVENT RONDO 100microgram inhaler |
| 75 | c13I. | AIROMIR 100micrograms CFC-free inhaler |
| 76 | c13j. | SALBUVENT 5mg/mL respirator solution |
| 77 | c13J. | SALBUTAMOL 100micrograms CFC-free inhaler |
| 78 | c13k. | *SALBUVENT RONDO spacer x1 |
| 79 | c13K. | SALAMOL EASI-BREATHE 100micrograms breath-actuated aerosol inhaler |
| 80 | c13l. | AEROLIN 100micrograms Autohaler 200d |
| 81 | c13L. | SALBUTAMOL 200micrograms breath-actuated dry powder inhaler |
| 82 | c13m. | VENTOLIN 5mg/2.5mL Nebules |
| 83 | c13M. | VENTOLIN 200micrograms Accuhaler |
| 84 | c13n. | AEROLIN 100micrograms Autohaler 100d |
| 85 | c13N. | SALBUTAMOL 100micrograms vortex metered dose inhaler |
| 86 | c13o. | SALBUTAMOL 5mg/2.5mL nebulisation units |
| 87 | c13O. | VENTOLIN EASI-BREATHE 100microgram inhaler |
| 88 | c13p. | *MAXIVENT 100microgram inhaler |
| 89 | c13P. | SALBUTAMOL 100micrograms Spacehaler |
| 90 | c13q. | *SALBUTAMOL 200 cyclocaps |
| 91 | c13Q. | ASMASAL 95micrograms Clickhaler |
| 92 | c13r. | *SALBUTAMOL 400 cyclocaps |
| 93 | c13R. | SALBUTAMOL 100micrograms breath-act dry powder inhaler |
| 94 | c13s. | *VENTOLIN rotahaler device |
| 95 | c13S. | SALBUTAMOL 95micrograms breath-actuated dry powder inhaler |
| 96 | c13T. | VENTOLIN 100micrograms Evohaler |
| 97 | c13U. | SALBUTAMOL 100micrograms CFC-free breath-actuated aerosol inhaler |
| 98 | c13v. | SALBUTAMOL 100microgram inhaler |
| 99 | c13V. | AIROMIR 100micrograms CFC-free Autohaler |
| 100 | c13w. | SALBUTAMOL 2.5mg/2.5mL nebulisation units |
| 101 | c13W. | MAXIVENT 2.5mg/2.5mL Steripoules |
| 102 | c13x. | SALBUTAMOL 200micrograms inhalation capsules |
| 103 | c13X. | MAXIVENT 5mg/2.5mL Steripoules |
| 104 | c13y. | SALBUTAMOL 400micrograms inhalation capsules |
| 105 | c13Y. | SALBULIN 100micrograms CFC-free inhaler |
| 106 | c13z. | SALBUTAMOL 100mg/20mL respirator solution |
| 107 | c13Z. | SALAMOL 100micrograms CFC-free inhaler |
| 108 | c14.. | TERBUTALINE SULFATE [RESPIRATORY USE] |
| 109 | c141. | BRICANYL 5mg tablets |
| **#** | **Read codes for asthma medications** | **Description** |
| 110 | c142. | BRICANYL 1.5mg/5mL syrup |
| 111 | c143. | BRICANYL 500micrograms/1mL injection |
| 112 | c144. | BRICANYL 250micrograms inhaler |
| 113 | c145. | BRICANYL 250micrograms refill cannister |
| 114 | c146. | BRICANYL 250micrograms spacer inhaler |
| 115 | c147. | BRICANYL RESPULES 5mg/2mL nebuliser solution |
| 116 | c148. | BRICANYL 100mg/10mL respirator solution |
| 117 | c149. | *BRICANYL SA 7.5mg m/r tablets |
| 118 | c14a. | *MONOVENT 5mg tablets |
| 119 | c14b. | *MONOVENT 1.5mg/5mL syrup |
| 120 | c14c. | *MONOVENT SA 7.5mg m/r tablets |
| 121 | c14d. | *NEBUHALER spacer device |
| 122 | c14e. | BRICANYL 2.5mg/5mL injection |
| 123 | c14f. | BRICANYL 500micrograms Turbohaler |
| 124 | c14g. | TERBUTALINE 500micrograms inhaler |
| 125 | c14h. | TERBUTALINE 2.5mg/5mL injection |
| 126 | c14i. | TERBUTALINE SULPHATE 200mg/20mL nebuliser solution |
| 127 | c14j. | TERBUTALINE 500micrograms breath-actuated dry powder inhaler |
| 128 | c14k. | BRICANYL 200mg/20mL respirator solution |
| 129 | c14r. | TERBUTALINE 5mg tablets |
| 130 | c14s. | TERBUTALINE 500microgram/1mL injection |
| 131 | c14t. | TERBUTALINE 250micrograms inhaler |
| 132 | c14u. | TERBUTALINE 250micrograms refill cannister |
| 133 | c14v. | TERBUTALINE 250micrograms spacer |
| 134 | c14w. | TERBUTALINE 5mg/2mL nebuliser solution |
| 135 | c14x. | TERBUTALINE 100mg/10mL respirator solution |
| 136 | c14y. | *TERBUTALINE 7.5mg m/r tablets |
| 137 | c14z. | TERBUTALINE 1.5mg/5mL syrup |
| 138 | c15.. | FENOTEROL HYDROBROMIDE |
| 139 | c151. | *BEROTEC 200micrograms inhaler |
| 140 | c152. | BEROTEC 100mg/20mL respirator solution |
| 141 | c153. | *BEROTEC 100micrograms inhaler |
| 142 | c154. | FENOTEROL 100micrograms inhaler |
| 143 | c15y. | FENOTEROL 200micrograms inhaler |
| 144 | c15z. | FENOTEROL 100mg/20mL respirator solution |
| 145 | c16.. | PIRBUTEROL |
| 146 | c161. | *EXIREL 10mg capsules |
| **#** | **Read codes for asthma medications** | **Description** |
| 147 | c162. | *EXIREL 15mg capsules |
| 148 | c163. | *EXIREL 7.5mg/5mL syrup |
| 149 | c164. | *EXIREL 200micrograms inhaler |
| 150 | c16w. | *PIRBUTEROL 10mg capsules |
| 151 | c16x. | *PIRBUTEROL 15mg capsules |
| 152 | c16y. | *PIRBUTEROL 7.5mg/5mL syrup |
| 153 | c16z. | PIRBUTEROL 200micrograms inhaler |
| 154 | c17.. | REPROTEROL HYDROCHLORIDE |
| 155 | c171. | *BRONCHODIL 20mg tablets |
| 156 | c172. | *BRONCHODIL 10mg/5mL elixir |
| 157 | c173. | BRONCHODIL 500micrograms inhaler |
| 158 | c174. | BRONCHODIL 10mg/mL respirator solution |
| 159 | c17w. | *REPROTEROL 20mg tablets |
| 160 | c17x. | *REPROTEROL 10mg/5mL elixir |
| 161 | c17y. | REPROTEROL 500micrograms inhaler |
| 162 | c17z. | REPROTEROL 10mg/mL respirator solution |
| 163 | c18.. | RIMITEROL HYDROBROMIDE |
| 164 | c181. | PULMADIL 200micrograms inhaler |
| 165 | c182. | PULMADIL 200micrograms autohaler |
| 166 | c183. | PULMADIL 200micrograms auto refill cannister |
| 167 | c184. | RIMITEROL 200micrograms breath-actuated aerosol inhaler |
| 168 | c18y. | RIMITEROL 200micrograms inhaler |
| 169 | c18z. | RIMITEROL 200micrograms auto refill cannister |
| 170 | c19.. | SALMETEROL XINAFOATE |
| 171 | c191. | SALMETEROL 25microgram inhaler |
| 172 | c192. | *SEREVENT 25microgram inhaler |
| 173 | c193. | *SEREVENT 50mcg diskhaler |
| 174 | c194. | SEREVENT 50micrograms disk refill |
| 175 | c195. | SALMETEROL 50micrograms disks+disk inhaler |
| 176 | c196. | SALMETEROL 50micrograms disk refill |
| 177 | c197. | SALMETEROL 50micrograms breath-actuated dry powder inhaler |
| 178 | c198. | SEREVENT 50micrograms Accuhaler |
| 179 | c199. | SEREVENT 25micrograms Evohaler |
| 180 | c19A. | NEOVENT 25micrograms CFC-free inhaler |
| 181 | c19B. | VERTINE 25micrograms CFC-free inhaler |
| 182 | c19z. | SALMETEROL 25micrograms CFC-free inhaler |
| 183 | c1a.. | TULOBUTEROL HYDROCHLORIDE |
| **#** | **Read codes for asthma medications** | **Description** |
| 184 | c1a1. | *TULOBUTEROL 2mg tablets |
| 185 | c1a2. | *BRELOMAX 2mg tablets |
| 186 | c1a3. | *RESPACAL 2mg tablets |
| 187 | c1a4. | TULOBUTEROL 1mg/5mL sugar free liquid |
| 188 | c1a5. | RESPACAL 1mg/5mL sugar free liquid |
| 189 | c1b.. | INDACATEROL |
| 190 | c1B.. | BAMBUTEROL HYDROCHLORIDE |
| 191 | c1b1. | ONBREZ BREEZHALER 150micrograms inhalation capsules+inhaler |
| 192 | c1B1. | BAMBEC 10mg tablets |
| 193 | c1b2. | INDACATEROL 150micrograms inhalation capsules+inhaler |
| 194 | c1B2. | BAMBEC 20mg tablets |
| 195 | c1b3. | ONBREZ BREEZHALER 300micrograms inhalation capsules+inhaler |
| 196 | c1B3. | BAMBUTEROL HYDROCHLORIDE 10mg tablets |
| 197 | c1b4. | INDACATEROL 300micrograms inhalation capsules+inhaler |
| 198 | c1B4. | BAMBUTEROL HYDROCHLORIDE 20mg tablets |
| 199 | c1c.. | FLUTICASONE PROPIONATE+FORMOTEROL FUMARATE |
| 200 | c1C.. | FORMOTEROL |
| 201 | c1c1. | FLUTIFORM 50micrograms/5micrograms inhaler |
| 202 | c1C1. | FORMOTEROL FUMARATE 12micrograms inhalation capsules+inhaler |
| 203 | c1c2. | FLUTIFORM 125micrograms/5micrograms inhaler |
| 204 | c1C2. | FORADIL 12micrograms inhalation capsules+inhaler |
| 205 | c1c3. | FLUTIFORM 250micrograms/10micrograms inhaler |
| 206 | c1C3. | FORMOTEROL FUMARATE DIHYDRATE 6micrograms breath-act dry powder inhaler |
| 207 | c1C4. | FORMOTEROL FUMARATE DIHYDRATE 12micrograms breath-act dry powder inhaler |
| 208 | c1C5. | OXIS 6micrograms Turbohaler |
| 209 | c1C6. | OXIS 12micrograms Turbohaler |
| 210 | c1C7. | ATIMOS MODULITE 12micrograms metered dose inhaler |
| 211 | c1C8. | FORMOTEROL EASYHALER 12micrograms breath-act dry powder inhaler |
| 212 | c1cx. | FLUTICASONE PROPIONATE+FORMOTEROL FUMARATE 250mcg/10mcg inh |
| 213 | c1cy. | FLUTICASONE PROPIONATE+FORMOTEROL FUMARATE 125mcg/5mcg inh |
| 214 | c1Cy. | FORMOTEROL FUMARATE DIHYDRATE 12micrograms breath-act dry powder inhaler |
| 215 | c1cz. | FLUTICASONE PROPIONATE+FORMOTEROL FUMARATE 50mcg/5mcg inh |
| 216 | c1Cz. | FORMOTEROL FUMARATE DIHYDRATE 12micrograms metered dose inhaler |
| 217 | c1d.. | OLODATEROL |
| **#** | **Read codes for asthma medications** | **Description** |
| 218 | c1D.. | SALMETEROL+FLUTICASONE PROPIONATE |
| 219 | c1d1. | STRIVERDI RESPIMAT 2.5micrograms inhaler |
| 220 | c1D1. | SERETIDE 100 Accuhaler |
| 221 | c1d2. | OLODATEROL 2.5micrograms inhaler |
| 222 | c1D2. | SERETIDE 250 Accuhaler |
| 223 | c1D3. | SERETIDE 500 Accuhaler |
| 224 | c1D4. | SERETIDE 50 Evohaler |
| 225 | c1D5. | SERETIDE 125 Evohaler |
| 226 | c1D6. | SERETIDE 250 Evohaler |
| 227 | c1D7. | SIRDUPLA 25micrograms/125micrograms inhaler |
| 228 | c1D8. | SIRDUPLA 25micrograms/250micrograms inhaler |
| 229 | c1D9. | AIRFLUSAL FORSPIRO 50micrograms/500micrograms pdr inhaler |
| 230 | c1Du. | SALMETEROL+FLUTICASONE PROPIONATE 25micrograms/50micrograms CFC-free inhaler |
| 231 | c1Dv. | SALMETEROL+FLUTICASONE PROPIONATE 25micrograms/125micrograms CFC-free inhaler |
| 232 | c1Dw. | SALMETEROL+FLUTICASONE PROPIONATE 25micrograms/250micrograms CFC-free inhaler |
| 233 | c1Dx. | SALMETEROL+FLUTICASONE PROPIONATE 50micrograms/100micrograms breath-actuated dry powder inhaler |
| 234 | c1Dy. | SALMETEROL+FLUTICASONE PROPIONATE 50micrograms/250micrograms breath-actuated dry powder inhaler |
| 235 | c1Dz. | SALMETEROL+FLUTICASONE PROPIONATE 50micrograms/500micrograms breath-actuated dry powder inhaler |
| 236 | c1e.. | INDACATEROL+GLYCOPYRRONIUM |
| 237 | c1E.. | SALBUTAMOL [INHALATION PREPRATIONS 2] |
| 238 | c1e1. | ULTIBRO BREEZHALER 85mcg/43mcg inh powder capsules+inhaler |
| 239 | c1E1. | SALAMOL EASI-BREATHE 100micrograms CFC-free breath-actuated aerosol inhaler |
| 240 | c1e2. | INDACATEROL+GLYCOPYRRONIUM 85mcg/43mcg inh powder caps+inh |
| 241 | c1E2. | PULVINAL SALBUTAMOL 200micrograms breath-actuated dry powder inhaler |
| 242 | c1E3. | VENTODISKS 200micrograms diskhaler 15x8 |
| 243 | c1E4. | VENTODISKS 400micrograms diskhaler 15x8 |
| 244 | c1E5. | VENTODISKS 200micrograms disk refill 15x8 |
| 245 | c1E6. | VENTODISKS 400micrograms disk refill 15x8 |
| 246 | c1E7. | EASYHALER SALBUTAMOL 100micrograms breath-actuated dry powder inhaler |
| 247 | c1E8. | EASYHALER SALBUTAMOL 200micrograms breath-actuated dry powder inhaler |
| **#** | **Read codes for asthma medications** | **Description** |
| 248 | c1E9. | SALBULIN NOVOLIZER 100micrograms dry powder cartridge and refillable inhaler device |
| 249 | c1EA. | SALBUTAMOL 100micrograms dry powder cartridge and refillable inhaler device |
| 250 | c1EB. | SALBULIN NOVOLIZER 100micrograms dry powder cartridge refill |
| 251 | c1EC. | SALBUTAMOL 100micrograms dry powder cartridge refill |
| 252 | c1ED. | VENTOLIN 50mg/10mL respirator solution |
| 253 | c1EE. | SALBUTAMOL 50mg/10mL respirator solution |
| 254 | c23.. | *ISOETHARINE HYDROCHLORIDE |
| 255 | c231. | *NUMOTAC 10mg m/r tablets |
| 256 | c23z. | ISOETHARINE HCL 10mg m/r tablets |
| 257 | c24.. | ISOPRENALINE SULFATE |
| 258 | c241. | *ALEUDRIN 20mg tablets |
| 259 | c242. | ALEUDRIN 1% spray for nebuliser |
| 260 | c243. | ISO-AUTOHALER 80microgram inhaler |
| 261 | c244. | ISO-AUTOHALER 80microgram inhaler |
| 262 | c245. | MEDIHALER-ISO 80micrograms inhaler |
| 263 | c246. | MEDIHALER-ISO FORTE 400micrograms inhaler |
| 264 | c24v. | ISOPRENALINE SULPHATE 20mg tablets |
| 265 | c24w. | ISOPRENALINE SULPHATE 1% spray for nebuliser |
| 266 | c24x. | ISOPRENALINE SULPHATE 80micrograms inhaler |
| 267 | c24y. | ISOPRENALINE SULPHATE 80micrograms inhaler refill |
| 268 | c24z. | ISOPRENALINE SULPHATE 400micrograms inhaler |
| 269 | c25.. | ORCIPRENALINE SULFATE [RESPIRATORY USE] |
| 270 | c251. | *ALUPENT 20mg tablets |
| 271 | c252. | *ALUPENT 10mg/5mL syrup |
| 272 | c253. | ALUPENT 500microgram/1mL injection |
| 273 | c254. | *ALUPENT 750micrograms inhaler |
| 274 | c255. | ALUPENT 750micrograms inhaler refill |
| 275 | c25v. | *ORCIPRENALINE 20mg tablets |
| 276 | c25w. | *ORCIPRENALINE 10mg/5mL syrup |
| 277 | c25x. | ORCIPRENALINE 500microgram/1mL injection |
| 278 | c25y. | ORCIPRENALINE 750micrograms inhaler |
| 279 | c25z. | ORCIPRENALINE 750micrograms inhaler refill |
| 280 | c3… | ANTICHOLINERGIC BRONCHODILATORS |
| 281 | c31.. | IPRATROPIUM BROMIDE [1] |
| 282 | c311. | *ATROVENT 20micrograms inhaler |
| 283 | c312. | ATROVENT 500microgram/2mL nebuliser solution |
| 284 | c313. | ATROVENT FORTE 40microgram inhaler |
| **#** | **Read codes for asthma medications** | **Description** |
| 285 | c314. | ATROVENT 250microgram/1mL nebuliser solution |
| 286 | c315. | ATROVENT 20micrograms Autohaler |
| 287 | c316. | STERI-NEB IPRATROPIUM 250micrograms/1mL nebulisation units |
| 288 | c317. | STERI-NEB IPRATROPIUM 500micrograms/2mL nebulisation units |
| 289 | c318. | ATROVENT 40micrograms Aerocaps refill pack |
| 290 | c319. | ATROVENT 40micrograms Aerocaps+Aerohaler device |
| 291 | c31A. | IPRATROPIUM BROMIDE 40mcg inhalation capsules |
| 292 | c31B. | IPRATROPIUM BROMIDE 40mcg inhalation capsules+inhaler device |
| 293 | c31C. | RESPONTIN 250micrograms/1mL Nebules |
| 294 | c31D. | RESPONTIN 500micrograms/2mL Nebules |
| 295 | c31E. | TROPIOVENT 250micrograms/1mL Steripoules |
| 296 | c31F. | TROPIOVENT 500micrograms/2mL Steripoules |
| 297 | c31G. | ATROVENT 20micrograms CFC-free inhaler |
| 298 | c31t. | IPRATROPIUM BROMIDE 20micrograms CFC-free inhaler |
| 299 | c31u. | IPRATROPIUM 20micrograms breath-actuated aerosol inhaler |
| 300 | c31v. | IPRATROPIUM 250micrograms/1mL nebuliser solution |
| 301 | c31w. | IPRATROPIUM 500micrograms/2mL nebuliser solution |
| 302 | c31x. | IPRATROPIUM 20micrograms inhaler |
| 303 | c31y. | IPRATROPIUM 250micrograms/mL nebuliser solution |
| 304 | c31z. | IPRATROPIUM 40microgram inhaler |
| 305 | c32.. | OXITROPIUM BROMIDE |
| 306 | c321. | OXITROPIUM 100micrograms/dose inhaler 200dose |
| 307 | c322. | OXIVENT 100micrograms/dose inhaler 200dose |
| 308 | c323. | OXIVENT 100micrograms Autohaler |
| 309 | c324. | OXITROPIUM 100micrograms breath-actuated aerosol inhaler |
| 310 | c33.. | TIOTROPIUM |
| 311 | c331. | TIOTROPIUM 18micrograms inhalation capsules |
| 312 | c332. | TIOTROPIUM 18micrograms capsules with inhaler device |
| 313 | c333. | TIOTROPIUM 2.5micrograms inhalation cartridges with inhaler device |
| 314 | c33x. | SPIRIVA RESPIMAT 2.5micrograms inhalation cartridges with Respimat inhaler device |
| 315 | c33y. | SPIRIVA COMBOPACK 18micrograms capsules with HandiHaler inhaler device |
| 316 | c33z. | SPIRIVA 18micrograms inhalation capsules |
| 317 | c34.. | ACLIDINIUM |
| 318 | c341. | EKLIRA GENUAIR 322micrograms/dose dry powder inhaler |
| 319 | c342. | ACLIDINIUM 322micrograms/dose dry powder inhaler |
| 320 | c35.. | UMECLIDINIUM |
| 321 | c351. | INCRUSE ELLIPTA 55micrograms/dose dry powder inhaler |
| **#** | **Read codes for asthma medications** | **Description** |
| 322 | c352. | UMECLIDINIUM 55micrograms/dose dry powder inhaler |
| 323 | c4… | XANTHINE BRONCHODILATORS |
| 324 | c41.. | AMINOPHYLLINE |
| 325 | c411. | AMINOPHYLLINE 100mg tablets |
| 326 | c412. | AMINOPHYLLINE 250mg/10mL injection |
| 327 | c413. | AMINOPHYLLINE 500mg/2mL injection |
| 328 | c414. | AMINOPHYLLINE 50mg suppositories |
| 329 | c415. | AMINOPHYLLINE 100mg suppositories |
| 330 | c416. | AMINOPHYLLINE 150mg suppositories |
| 331 | c417. | AMINOPHYLLINE 180mg suppositories |
| 332 | c418. | AMINOPHYLLINE 360mg suppositories |
| 333 | c419. | *THEODROX tablets |
| 334 | c41a. | PHYLLOCONTIN CONTINUS 225mg m/r tablets |
| 335 | c41A. | *NORPHYLLIN 100mg tablets |
| 336 | c41b. | PHYLLOCONTIN FORTE 350mg m/r tablets |
| 337 | c41B. | NORPHYLLIN SR 225mg m/r tablets |
| 338 | c41c. | PHYLLOCONTIN PAEDIATRIC 100mg m/r tablets |
| 339 | c41C. | NORPHYLLIN SR 350mg m/r tablets |
| 340 | c41d. | AMINOPHYLLINE 225mg m/r tablets |
| 341 | c41e. | *PECRAM 225mg m/r tablets |
| 342 | c41f. | AMINOPHYLLINE 350mg m/r tablets |
| 343 | c41g. | AMINOPHYLLINE 100mg m/r tablets |
| 344 | c41h. | *AMNIVENT 225mg m/r tablets |
| 345 | c41i. | *AMNIVENT 350mg m/r tablets |
| 346 | c41j. | MIN-I-JET AMINOPHYLLINE 250mg/10mL injection |
| 347 | c41k. | AMINOPHYLLINE 250mg/10mL prefilled syringe |
| 348 | c41m. | AMINOPHYLLINE HYDRATE 225mg m/r tablets |
| 349 | c42.. | CHOLINE THEOPHYLLINATE |
| 350 | c421. | *CHOLEDYL 100mg tablets |
| 351 | c422. | *CHOLEDYL 200mg tablets |
| 352 | c423. | *CHOLEDYL 62.5mg/5mL syrup |
| 353 | c424. | SABIDAL SR-270 424mg m/r tablets |
| 354 | c42w. | CHOLINE THEOPHYLLINATE 100mg tablets |
| 355 | c42x. | CHOLINE THEOPHYLLINATE 200mg tablets |
| 356 | c42y. | CHOLINE THEOPHYLLINATE 62.5mg/5mL syrup |
| 357 | c42z. | CHOLINE THEOPHYLLINATE 424mg m/r tablets |
| 358 | c43.. | THEOPHYLLINE |
| **#** | **Read codes for asthma medications** | **Description** |
| 359 | c431. | *BIOPHYLLINE 125mg/5mL syrup |
| 360 | c432. | *NUELIN 125mg tablets |
| 361 | c433. | *NUELIN 60mg/5mL liquid |
| 362 | c434. | *LASMA 300mg m/r tablets |
| 363 | c435. | NUELIN SA 175mg m/r tablets |
| 364 | c436. | NUELIN SA-250 250mg m/r tablets |
| 365 | c437. | *PRO-VENT 300mg m/r capsules |
| 366 | c438. | SLO-PHYLLIN 60mg m/r capsules |
| 367 | c439. | SLO-PHYLLIN 125mg m/r capsules |
| 368 | c43a. | SLO-PHYLLIN 250mg m/r capsules |
| 369 | c43A. | THEOPHYLLINE 200mg/10mL injection |
| 370 | c43b. | *THEO-DUR 200mg m/r tablets |
| 371 | c43B. | THEOPHYLLINE 10mg/5mL sugar free solution |
| 372 | c43c. | *THEO-DUR 300mg m/r tablets |
| 373 | c43d. | *THEOGRAD 350mg m/r tablets |
| 374 | c43e. | UNIPHYLLIN CONTINUS 400mg m/r tablets |
| 375 | c43f. | UNIPHYLLIN CONTINUS 200mg m/r tablets |
| 376 | c43g. | LABOPHYLLINE 200mg/10mL injection |
| 377 | c43h. | UNIPHYLLIN CONTINUS 300mg m/r tablets |
| 378 | c43i. | *BIOPHYLLINE 350mg m/r tablets |
| 379 | c43j. | *BIOPHYLLINE 500mg m/r tablets |
| 380 | c43k. | THEOPHYLLINE 500mg m/r tablets |
| 381 | c43m. | *THEOPHYLLINE 125mg/5mL syrup |
| 382 | c43n. | *THEOPHYLLINE 125mg tablets |
| 383 | c43o. | *THEOPHYLLINE 60mg/5mL liquid |
| 384 | c43p. | THEOPHYLLINE 175mg m/r tablets |
| 385 | c43q. | THEOPHYLLINE 250mg m/r tablets |
| 386 | c43r. | THEOPHYLLINE 300mg m/r capsules |
| 387 | c43s. | THEOPHYLLINE 60mg m/r capsules |
| 388 | c43t. | THEOPHYLLINE 125mg m/r capsules |
| 389 | c43u. | THEOPHYLLINE 250mg m/r capsules |
| 390 | c43v. | THEOPHYLLINE 200mg m/r tablets |
| 391 | c43w. | THEOPHYLLINE 300mg m/r tablets |
| 392 | c43x. | THEOPHYLLINE 350mg m/r tablets |
| 393 | c43y. | THEOPHYLLINE 400mg m/r tablets |
| 394 | c43z. | *THEOPHYLLINE 200mg tablets |
| 395 | c51.. | COMPOUND BRONCHODILATORS A-Z |
| **#** | **Read codes for asthma medications** | **Description** |
| 396 | c512. | ALUPENT EXPECTORANT 20mg tablets |
| 397 | c513. | *ALUPENT EXPECTORANT mixture |
| 398 | c516. | *BRICANYL COMPOUND tablets |
| 399 | c517. | *BRICANYL EXPECTORANT elixir |
| 400 | c51A. | FENOTEROL HYDROBROM+IPRATROPIUM BR 100micrograms/40micrograms inhaler |
| 401 | c51B. | FENOTEROL HYDROBROM+IPRATROPIUM BR 100micrograms/40micrograms breath-act aerosol inhaler |
| 402 | c51C. | SALBUTAMOL+IPRATROPIUM BROMIDE 100micrograms/20micrograms inhaler |
| 403 | c51D. | *COMBIVENT inhaler |
| 404 | c51E. | COMBIVENT Unit Dose Vials |
| 405 | c51F. | SALBUTAMOL+IPRATROPIUM BROMIDE 2.5mg/500micrograms nebulisation units |
| 406 | c51G. | SALIPRANEB nebuliser solution 2.5mL |
| 407 | c51g. | *DUO-AUTOHALER inhaler |
| 408 | c51H. | IPRATROPIUM BROMIDE+SALBUTAMOL 500mcg/2.5mg nebuliser soln |
| 409 | c51h. | DUO-AUTOHALER refill cannister |
| 410 | c51I. | ANORO ELLIPTA 55micrograms/22micrograms dry powder inhaler |
| 411 | c51i. | *DUOVENT inhaler |
| 412 | c51j. | ISO-BROVON pressurised inhaler |
| 413 | c51J. | UMECLIDINIUM+VILANTEROL 55mcg/22mcg dry powder inhaler |
| 414 | c51k. | ISO-BROVON PLUS pressurised inhaler |
| 415 | c51K. | DUAKLIR GENUAIR 340micrograms/12micrograms powder inhaler |
| 416 | c51l. | *MEDIHALER DUO inhaler |
| 417 | c51L. | ACLIDINIUM+FORMOTEROL FUMARATE DIHYD 340mcg/12mcg pdr inh |
| 418 | c51m. | *NETHAPRIN DOSPAN m/r tablets |
| 419 | c51M. | SPIOLTO RESPIMAT 2.5micrograms/2.5micrograms inhaler |
| 420 | c51N. | TIOTROPIUM+OLODATEROL 2.5micrograms/2.5micrograms inhaler |
| 421 | c51o. | *RYBAR NO-1 inhaler |
| 422 | c51p. | *RYBAR NO-2 inhaler |
| 423 | c51q. | *TAUMASTHMAN tablets |
| 424 | c51r. | *TEDRAL tablets |
| 425 | c51s. | *TEDRAL elixir |
| 426 | c51t. | *FRANOL NEW 11mg/120mg tablets |
| 427 | c51u. | FRANOL PLUS NEW 15mg/120mg tablets |
| 428 | c51v. | DUOVENT UDV nebuliser solution 4mL |
| **#** | **Read codes for asthma medications** | **Description** |
| 429 | c51w. | IPRATROPIUM BR+FENOTEROL HBR 500micrograms/1.25mg nebuliser solution 4mL |
| 430 | c51x. | *DUOVENT Autohaler |
| 431 | c531. | IPRAMOL STERI-NEB 2.5mg/500micrograms nebuliser solution 2.5mL |
| 432 | c6… | CORTICOSTEROIDS [RESPIRATORY USE] |
| 433 | c61.. | BECLOMETASONE DIPROPIONATE [RESPIRATORY USE] |
| 434 | c611. | BECLOFORTE 250microgram inhaler |
| 435 | c612. | BECOTIDE-50 50microgram inhaler |
| 436 | c613. | BECOTIDE 100micrograms rotacaps |
| 437 | c614. | BECOTIDE 200micrograms rotacaps |
| 438 | c615. | *BECOTIDE rotahaler device |
| 439 | c616. | BECOTIDE 50micrograms/mL nebuliser solution |
| 440 | c617. | BECOTIDE-100 100microgram inhaler |
| 441 | c618. | *VOLUMATIC spacer device |
| 442 | c619. | BECODISK 100micrograms diskhaler 14x8 |
| 443 | c61a. | BECODISK 200micrograms diskhaler 14x8 |
| 444 | c61A. | BECLOMETASONE DIPROPIONATE 400micrograms disks+disk inhaler |
| 445 | c61b. | BECOTIDE 400micrograms rotacaps |
| 446 | c61B. | BECLOMETASONE DIPROPIONATE 400micrograms disk refill |
| 447 | c61c. | BECODISK 100micrograms disk refill 14x8 |
| 448 | c61C. | BECLOMETHASONE DIPROPIONATE 250micrograms inhaler+spacer device |
| 449 | c61d. | BECODISK 200micrograms disk refill 14x8 |
| 450 | c61D. | BECLOMETASONE DIPROPIONATE 50micrograms breath-actuated aerosol inhaler |
| 451 | c61e. | BECODISK 400micrograms diskhaler 7x8 |
| 452 | c61E. | BECLOMETASONE DIPROPIONATE 250micrograms breath-actuated aerosol inhaler |
| 453 | c61f. | BECODISK 400micrograms disk refill 7x8 |
| 454 | c61F. | BECLOMETASONE DIPROPIONATE 100micrograms breath-actuated aerosol inhaler |
| 455 | c61g. | BECLOFORTE VM 250micrograms inhaler+volumatic |
| 456 | c61G. | *FILAIR 50micrograms inhaler |
| 457 | c61h. | BECLOMETASONE DIPROPIONATE 400micrograms inhalation capsules |
| 458 | c61H. | *FILAIR 100micrograms inhaler |
| 459 | c61i. | BECOTIDE-200 200microgram inhaler |
| 460 | c61j. | *AEROBEC 50microgram Autohaler |
| 461 | c61J. | FILAIR FORTE 250micrograms inhaler |
| 462 | c61k. | AEROBEC FORTE 250micrograms Autohaler |
| 463 | c61K. | BECLAZONE 50micrograms inhaler |
| **#** | **Read codes for asthma medications** | **Description** |
| 464 | c61l. | AEROBEC 100microgram Autohaler |
| 465 | c61L. | BECLAZONE 100micrograms inhaler |
| 466 | c61m. | BECLOFORTE DISKHALER 400micrograms 14x8 |
| 467 | c61M. | BECLAZONE 250micrograms inhaler |
| 468 | c61n. | BECLOFORTE DISKS 400micrograms disk refill 14x8 |
| 469 | c61N. | BECLAZONE 50 EASI-BREATHE inhaler |
| 470 | c61O. | BECLAZONE 100 EASI-BREATHE inhaler |
| 471 | c61p. | BECLOMETASONE DIPROPIONATE 100micrograms disks+disk inhaler |
| 472 | c61P. | BECLAZONE 250 EASI-BREATHE inhaler |
| 473 | c61q. | BECLOMETASONE DIPROPIONATE 200micrograms disks+disk inhaler |
| 474 | c61Q. | BECLOFORTE INTEGRA 250micrograms inhaler+compact spacer |
| 475 | c61r. | BECLOMETASONE DIPROPIONATE 100micrograms disk refill |
| 476 | c61R. | BECLOFORTE INTEGRA 250micrograms refill |
| 477 | c61s. | BECLOMETASONE DIPROPIONATE 200micrograms disk refill |
| 478 | c61S. | BECLOMETHASONE DIPROPIONATE 250micrograms inhaler+compact spacer |
| 479 | c61t. | BECLOMETASONE DIPROPIONATE 250micrograms inhaler |
| 480 | c61T. | BECLOMETHASONE DIPROPIONATE 250micrograms compact spacer refill |
| 481 | c61u. | BECLOMETASONE DIPROPIONATE 200micrograms inhaler |
| 482 | c61U. | BECLOMETHASONE rotahaler device |
| 483 | c61v. | BECLOMETASONE DIPROPIONATE 50micrograms inhaler |
| 484 | c61V. | BECLOMETHASONE DIPROPIONATE 50micrograms vortex metered dose inhaler |
| 485 | c61w. | BECLOMETASONE DIPROPIONATE 100micrograms inhalation capsules |
| 486 | c61W. | *BDP 50micrograms Spacehaler |
| 487 | c61x. | BECLOMETASONE DIPROPIONATE 200micrograms inhalation capsules |
| 488 | c61X. | BECLOMETHASONE DIPROPIONATE 100micrograms vortex metered dose inhaler |
| 489 | c61y. | BECLOMETHASONE DIPROPIONATE 50micrograms/mL nebuliser solution |
| 490 | c61Y. | *BDP 100micrograms Spacehaler |
| 491 | c61z. | BECLOMETASONE DIPROPIONATE 100micrograms inhaler |
| 492 | c61Z. | BECLOMETHASONE DIPROPIONATE 250micrograms vortex metered dose inhaler |
| 493 | c62.. | BECLOMETASONE COMPOUNDS |
| 494 | c621. | *VENTIDE inhaler |
| 495 | c622. | *VENTIDE Rotacaps |
| 496 | c623. | *VENTIDE paediatric Rotacaps |
| 497 | c624. | *VENTIDE Rotahaler device |
| 498 | c63.. | *BETAMETHASONE VALERATE |
| 499 | c631. | *BEXTASOL 100microgram inhaler |
| 500 | c63z. | BETAMETHASONE 100micrograms inhaler |
| **#** | **Read codes for asthma medications** | **Description** |
| 501 | c64.. | BUDESONIDE [RESPIRATORY USE] |
| 502 | c641. | PULMICORT 200micrograms inhaler 200dose |
| 503 | c642. | PULMICORT 200micrograms refill 100dose |
| 504 | c643. | PULMICORT 200micrograms refill 200dose |
| 505 | c644. | PULMICORT LS 50micrograms inhaler |
| 506 | c645. | PULMICORT LS 50micrograms refill |
| 507 | c646. | *NEBUHALER spacer device |
| 508 | c647. | PULMICORT 200microgram inhaler 100dose |
| 509 | c648. | PULMICORT 200microgram Turbohaler 100dose |
| 510 | c649. | PULMICORT 400microgram Turbohaler 50dose |
| 511 | c64a. | PULMICORT 500micrograms Respules 2mL unit |
| 512 | c64A. | BUDESONIDE 200micrograms refill cannister |
| 513 | c64b. | PULMICORT 1mg Respules 2mL unit |
| 514 | c64B. | BUDESONIDE 50micrograms spacer inhaler |
| 515 | c64c. | PULMICORT 100microgram Turbohaler 200dose |
| 516 | c64C. | PULMICORT 200micrograms spacer inhaler |
| 517 | c64d. | BUDESONIDE 100micrograms breath-actuated dry powder inhaler |
| 518 | c64D. | PULMICORT LS 50micrograms spacer inhaler |
| 519 | c64e. | BUDESONIDE 50micrograms refill cannister |
| 520 | c64E. | PULMICORT 200micrograms inhaler with NebuChamber |
| 521 | c64F. | BUDESONIDE 200micrograms/dose dry powder cartridge refill |
| 522 | c64g. | BUDESONIDE 200micrograms breath-actuated dry powder inhaler |
| 523 | c64G. | NOVOLIZER BUDESONIDE 200micrograms/dose dry powder cartridge refill |
| 524 | c64h. | BUDESONIDE 400micrograms breath-actuated dry powder inhaler |
| 525 | c64H. | EASYHALER BUDESONIDE 100micrograms breath-actuated dry powder inhaler |
| 526 | c64i. | BUDESONIDE 500micrograms/2mL nebuliser solution |
| 527 | c64I. | EASYHALER BUDESONIDE 200micrograms breath-actuated dry powder inhaler |
| 528 | c64j. | BUDESONIDE 1mg/2mL nebuliser solution |
| 529 | c64J. | EASYHALER BUDESONIDE 400micrograms breath-actuated dry powder inhaler |
| 530 | c64k. | *BUDESONIDE 200 Cyclocaps |
| 531 | c64K. | PULMICORT 100micrograms CFC-free inhaler |
| 532 | c64l. | *BUDESONIDE 400 Cyclocaps |
| 533 | c64L. | BUDESONIDE 100micrograms CFC-free inhaler |
| 534 | c64m. | BUDESONIDE 200micrograms inhalation capsules |
| 535 | c64M. | PULMICORT 200micrograms CFC-free inhaler |
| 536 | c64n. | BUDESONIDE 400micrograms inhalation capsules |
| 537 | c64N. | BUDESONIDE 200micrograms CFC-free inhaler |
| **#** | **Read codes for asthma medications** | **Description** |
| 538 | c64o. | BUDESONIDE 200micrograms inhaler with spacer device |
| 539 | c64p. | NOVOLIZER BUDESONIDE 200micrograms/dose dry powder cartridge and refillable inhaler device |
| 540 | c64u. | BUDESONIDE 200micrograms/dose dry powder cartridge and refillable inhaler device |
| 541 | c64v. | BUDESONIDE 200micrograms inhaler |
| 542 | c64w. | *BUDESONIDE refill 100dose |
| 543 | c64x. | *BUDESONIDE refill 200dose |
| 544 | c64y. | BUDESONIDE 50micrograms inhaler |
| 545 | c64z. | BUDESONIDE 200micrograms spacer inhaler |
| 546 | c65.. | FLUTICASONE PROPIONATE [RESPIRATORY USE] |
| 547 | c651. | FLIXOTIDE 50micrograms diskhaler |
| 548 | c652. | FLIXOTIDE 100micrograms diskhaler |
| 549 | c653. | FLIXOTIDE 250micrograms diskhaler |
| 550 | c654. | FLUTICASONE PROPIONATE 50micrograms disks+disk inhaler |
| 551 | c655. | FLUTICASONE PROPIONATE 100micrograms disks+disk inhaler |
| 552 | c656. | FLUTICASONE PROPIONATE 250micrograms disks+disk inhaler |
| 553 | c657. | FLIXOTIDE 50micrograms disk refill |
| 554 | c658. | FLIXOTIDE 100micrograms disk refill |
| 555 | c659. | FLIXOTIDE 250micrograms disk refill |
| 556 | c65a. | FLIXOTIDE 2mg/2mL Nebules |
| 557 | c65A. | FLUTICASONE PROPIONATE 50micrograms disk refill |
| 558 | c65b. | FLUTICASONE PROPIONATE 125micrograms CFC-free inhaler |
| 559 | c65B. | FLUTICASONE PROPIONATE 100micrograms disk refill |
| 560 | c65c. | FLUTICASONE PROPIONATE 250micrograms CFC-free inhaler |
| 561 | c65C. | FLUTICASONE PROPIONATE 250micrograms disk refill |
| 562 | c65d. | FLIXOTIDE 125micrograms Evohaler |
| 563 | c65D. | FLIXOTIDE 25micrograms inhaler |
| 564 | c65e. | FLIXOTIDE 250micrograms Evohaler |
| 565 | c65E. | FLIXOTIDE 50micrograms inhaler |
| 566 | c65f. | FLUTICASONE PROPIONATE 50micrograms CFC-free inhaler |
| 567 | c65F. | FLIXOTIDE 125micrograms inhaler |
| 568 | c65g. | FLIXOTIDE 50micrograms Evohaler |
| 569 | c65G. | FLUTICASONE PROPIONATE 25micrograms inhaler |
| 570 | c65H. | FLUTICASONE PROPIONATE 50micrograms inhaler |
| 571 | c65I. | FLUTICASONE PROPIONATE 125micrograms inhaler |
| 572 | c65J. | FLUTICASONE PROPIONATE 250micrograms inhaler |
| **#** | **Read codes for asthma medications** | **Description** |
| 573 | c65K. | FLIXOTIDE 250micrograms inhaler |
| 574 | c65L. | FLIXOTIDE 500micrograms diskhaler |
| 575 | c65M. | FLIXOTIDE 500micrograms disk refill |
| 576 | c65N. | FLUTICASONE PROPIONATE 500micrograms disks+disk inhaler |
| 577 | c65O. | FLUTICASONE PROPIONATE 500micrograms disk refill |
| 578 | c65P. | FLUTICASONE PROPIONATE 50micrograms breath-actuated dry powder inhaler |
| 579 | c65Q. | FLUTICASONE PROPIONATE 100micrograms breath-actuated dry powder inhaler |
| 580 | c65R. | FLUTICASONE PROPIONATE 250micrograms breath-actuated dry powder inhaler |
| 581 | c65S. | FLUTICASONE PROPIONATE 500micrograms breath-actuated dry powder inhaler |
| 582 | c65T. | FLIXOTIDE 50micrograms Accuhaler |
| 583 | c65U. | FLIXOTIDE 100micrograms Accuhaler |
| 584 | c65V. | FLIXOTIDE 250micrograms Accuhaler |
| 585 | c65W. | FLIXOTIDE 500micrograms Accuhaler |
| 586 | c65X. | FLUTICASONE PROPIONATE 0.5mg/2mL nebulisation units |
| 587 | c65Y. | FLUTICASONE PROPIONATE 2mg/2mL nebulisation units |
| 588 | c65Z. | FLIXOTIDE 0.5mg/2mL Nebules |
| 589 | c66.. | BECLOMETASONE DIPROPIONATE [RESPIRATORY USE 2] |
| 590 | c661. | *BDP 250micrograms Spacehaler |
| 591 | c662. | BECOTIDE 50 EASI-BREATHE inhaler |
| 592 | c663. | BECOTIDE 100 EASI-BREATHE inhaler |
| 593 | c664. | BECLOFORTE EASI-BREATHE 250micrograms inhaler |
| 594 | c665. | QVAR 50 inhaler |
| 595 | c666. | QVAR 100 inhaler |
| 596 | c667. | QVAR 50 Autohaler |
| 597 | c668. | QVAR 100 Autohaler |
| 598 | c669. | *BECLAZONE 200 inhaler |
| 599 | c66a. | QVAR EASI-BREATHE 100micrograms CFC-free breath-actuated dry powder inhaler |
| 600 | c66A. | BECLOMETASONE DIPROPIONATE 50micrograms breath-actuated dry powder inhaler |
| 601 | c66b. | EASYHALER BECLOMETASONE 200micrograms breath-actuated dry powder inhaler |
| 602 | c66B. | BECLOMETASONE DIPROPIONATE 100micrograms breath-actuated dry powder inhaler |
| 603 | c66c. | CLENIL MODULITE 50micrograms CFC-free inhaler |
| 604 | c66C. | BECLOMETASONE DIPROPIONATE 250micrograms breath-actuated dry powder inhaler |
| 605 | c66d. | CLENIL MODULITE 100micrograms CFC-free inhaler |
| 606 | c66D. | ASMABEC 50micrograms Clickhaler |
| **#** | **Read codes for asthma medications** | **Description** |
| 607 | c66e. | CLENIL MODULITE 200micrograms CFC-free inhaler |
| 608 | c66E. | ASMABEC 100micrograms Clickhaler |
| 609 | c66f. | CLENIL MODULITE 250micrograms CFC-free inhaler |
| 610 | c66F. | ASMABEC 250micrograms Clickhaler |
| 611 | c66g. | BECLOMETASONE DIPROPIONATE 200micrograms CFC-free inhaler |
| 612 | c66G. | BECLOMETASONE DIPROPIONATE 400micrograms breath-actuated dry powder inhaler |
| 613 | c66h. | BECLOMETASONE DIPROPIONATE 250micrograms CFC-free inhaler |
| 614 | c66H. | BECLOMETASONE DIPROPIONATE 200micrograms breath-actuated dry powder inhaler |
| 615 | c66I. | PULVINAL BECLOMETHASONE DIPROPIONATE 100micrograms breath-actuated dry powder inhaler |
| 616 | c66J. | PULVINAL BECLOMETHASONE DIPROPIONATE 200micrograms breath-actuated dry powder inhaler |
| 617 | c66K. | PULVINAL BECLOMETHASONE DIPROPIONATE 400micrograms breath-actuated dry powder inhaler |
| 618 | c66L. | *BECLOMETASONE 100 cyclocaps |
| 619 | c66M. | *BECLOMETASONE 200 cyclocaps |
| 620 | c66N. | *BECLOMETASONE 400 cyclocaps |
| 621 | c66P. | BECODISK 100micrograms diskhaler 15x8 |
| 622 | c66Q. | BECODISK 200micrograms diskhaler 15x8 |
| 623 | c66R. | BECODISK 400micrograms diskhaler 15x8 |
| 624 | c66S. | BECODISK 100micrograms disk refill 15x8 |
| 625 | c66T. | BECODISK 200micrograms disk refill 15x8 |
| 626 | c66U. | BECODISK 400micrograms disk refill 15x8 |
| 627 | c66V. | BECLOMETASONE DIPROPIONATE 50micrograms CFC-free inhaler |
| 628 | c66W. | BECLOMETASONE DIPROPIONATE 100micrograms CFC-free inhaler |
| 629 | c66X. | BECLOMETASONE DIPROPIONATE 50micrograms CFC-free breath-actuated aerosol inhaler |
| 630 | c66Y. | BECLOMETASONE DIPROPIONATE 100micrograms CFC-free breath-actuated aerosol inhaler |
| 631 | c66Z. | QVAR EASI-BREATHE 50micrograms CFC-free breath-actuated dry powder inhaler |
| 632 | c67.. | BUDESONIDE+FORMOTEROL |
| 633 | c671. | SYMBICORT 100/6 Turbohaler |
| 634 | c672. | SYMBICORT 200/6 Turbohaler |
| 635 | c673. | SYMBICORT 400/12 Turbohaler |
| 636 | c674. | DUORESP SPIROMAX 160mcg/4.5mcg breath-act dry powder inhaler |
| 637 | c675. | DUORESP SPIROMAX 320mcg/9mcg breath-act dry powder inhaler |
| **#** | **Read codes for asthma medications** | **Description** |
| 638 | c67x. | BUDESONIDE+FORMOTEROL FUMARATE DIHYDRATE 400micrograms/12micrograms breath-actuated dry powder inhaler |
| 639 | c67y. | BUDESONIDE+FORMOTEROL FUMARATE DIHYDRATE 200micrograms/6micrograms breath-actuated dry powder inhaler |
| 640 | c67z. | BUDESONIDE+FORMOTEROL FUMARATE DIHYDRATE 100micrograms/6micrograms breath-actuated dry powder inhaler |
| 641 | c68.. | MOMETASONE [RESPIRATORY USE] |
| 642 | c681. | MOMETASONE FUROATE 200micrograms breath-actuated dry powder inhaler |
| 643 | c682. | MOMETASONE FUROATE 400micrograms breath-actuated dry powder inhaler |
| 644 | c683. | ASMANEX TWISTHALER 200micrograms breath-actuated dry powder inhaler |
| 645 | c684. | ASMANEX TWISTHALER 400micrograms breath-actuated dry powder inhaler |
| 646 | c69.. | CICLESONIDE |
| 647 | c691. | ALVESCO 160micrograms inhaler |
| 648 | c692. | ALVESCO 80micrograms inhaler |
| 649 | c69y. | CICLESONIDE 80micrograms inhaler |
| 650 | c69z. | CICLESONIDE 160micrograms inhaler |
| 651 | c6A.. | BECLOMETASONE+FORMOTEROL |
| 652 | c6A1. | FOSTAIR 100micrograms/6micrograms inhaler |
| 653 | c6A2. | FOSTAIR NEXTHALER 100micrograms/6micrograms powder inhaler |
| 654 | c6A3. | FOSTAIR 200micrograms/6micrograms inhaler |
| 655 | c6A4. | FOSTAIR NEXTHALER 200micrograms/6micrograms powder inhaler |
| 656 | c6Aw. | BECLOMET DIPROP+FORMOTERL FUMARATE DIHYD 200mcg/6mcg pdr inh |
| 657 | c6Ax. | BECLOMET DIPROP+FORMOTERL FUMARATE DIHYD 200mcg/6mcg inhaler |
| 658 | c6Ay. | BECLOMET DIPROP+FORMOTERL FUMARATE DIHYD 100mcg/6mcg pdr inh |
| 659 | c6Az. | BECLOMETASONE DIPROPIONATE+FORMOTEROL FUMARATE DIHYDRATE 100micrograms/6micrograms inhaler |
| 660 | c6B.. | FLUTICASONE+VILANTEROL |
| 661 | c6B1. | RELVAR ELLIPTA 184micrograms/22micrograms inhaler |
| 662 | c6B2. | FLUTICASONE FUROATE+VILANTEROL 184mcg/22mcg dry pdr inhaler |
| 663 | c6B3. | RELVAR ELLIPTA 92micrograms/22micrograms inhaler |
| 664 | c6B4. | FLUTICASONE FUROATE+VILANTEROL 92mcg/22mcg dry pdr inhaler |
| 665 | c7… | ASTHMA PROPHYLAXIS |
| 666 | c71.. | SODIUM CROMOGLICATE [ASTHMA] |
| 667 | c711. | *INTAL 1mg inhaler |
| 668 | c712. | *INTAL HALERMATIC insufflator |
| 669 | c713. | *INTAL 20mg spincaps |
| 670 | c714. | *INTAL SPINHALER insufflator |
| **#** | **Read codes for asthma medications** | **Description** |
| 671 | c715. | INTAL 20mg/2mL nebuliser solution |
| 672 | c716. | *INTAL 5mg inhaler |
| 673 | c717. | SODIUM CROMOGLICATE 20mg inhalation capsules |
| 674 | c718. | SODIUM CROMOGLICATE 20mg/2mL nebuliser solution |
| 675 | c719. | SODIUM CROMOGLICATE 5mg inhaler |
| 676 | c71a. | *INTAL 5mg Autohaler |
| 677 | c71b. | STERI-NEB CROMOGEN 20mg nebulisation units |
| 678 | c71c. | *CROMOGEN 5mg inhaler |
| 679 | c71d. | INTAL FISONAIR 5mg inhaler + spacer device |
| 680 | c71e. | SODIUM CROMOGLICATE 5mg inhaler + spacer device |
| 681 | c71f. | SODIUM CROMOGLYCATE 5mg auto inhaler |
| 682 | c71g. | INTAL SYNCRONER 5mg inhaler + spacer device 2x112dose |
| 683 | c71h. | SODIUM CROMOGLICATE 5mg breath-actuated aerosol inhaler |
| 684 | c71i. | INTAL 5mg CFC-free inhaler |
| 685 | c71j. | CROMOGEN EASI-BREATHE 5mg breath-actuated aerosol inhaler |
| 686 | c71k. | SODIUM CROMOGLICATE 5mg CFC-free inhaler |
| 687 | c72.. | SODIUM CROMOGLICATE COMPOUNDS |
| 688 | c721. | *INTAL COMPOUND spincaps |
| 689 | c722. | *AEROCROM inhaler |
| 690 | c723. | AEROCROM SYNCRONER inhaler + spacer device |
| 691 | c72y. | SODIUM CROMOGLICATE+SALBUTAMOL 1mg/100micrograms inhaler + spacer device |
| 692 | c72z. | SODIUM CROMOGLICATE+SALBUTAMOL 1mg/100micrograms inhaler |
| 693 | c73.. | KETOTIFEN [ASTHMA PROPHYLAXIS] |
| 694 | c731. | *ZADITEN 1mg capsules |
| 695 | c732. | ZADITEN 1mg tablets |
| 696 | c733. | ZADITEN 1mg/5mL elixir |
| 697 | c734. | *KETOTIFEN 1mg capsules |
| 698 | c735. | KETOTIFEN 1mg tablets |
| 699 | c736. | KETOTIFEN 1mg/5mL elixir |
| 700 | c73x. | *KETOTIFEN 1mg capsules |
| 701 | c73y. | *KETOTIFEN 1mg tablets |
| 702 | c73z. | *KETOTIFEN 1mg/5mL elixir |
| 703 | c74.. | NEDOCROMIL SODIUM [ASTHMA] |
| 704 | c741. | *TILADE MINT 2mg inhaler |
| 705 | c742. | *NEDOCROMIL SODIUM 2mg inhaler |
| 706 | c743. | *TILADE MINT 2mg inhaler |
| **#** | **Read codes for asthma medications** | **Description** |
| 707 | c744. | TILADE MINT SYNCRONER 2mg inhaler |
| 708 | c745. | NEDOCROMIL SODIUM 2mg inhaler + spacer |
| 709 | c746. | NEDOCROMIL SODIUM 2mg CFC-free inhaler |
| 710 | c747. | TILADE 2mg CFC-free inhaler |
| 711 | c75.. | MEPOLIZUMAB |
| 712 | c751. | NUCALA 100mg powder for solution for injection |
| 713 | c752. | MEPOLIZUMAB 100mg powder for solution for injection |
| 714 | cA... | LEUKOTRIENE RECEPTOR ANTAGONIST |
| 715 | cA1.. | MONTELUKAST |
| 716 | cA11. | MONTELUKAST 10mg tablets |
| 717 | cA12. | MONTELUKAST 5mg chewable tablets |
| 718 | cA13. | SINGULAIR 10mg tablets |
| 719 | cA14. | SINGULAIR PAEDIATRIC 5mg chewable tablets |
| 720 | cA15. | SINGULAIR PAEDIATRIC 4mg chewable tablets |
| 721 | cA16. | SINGULAIR PAEDIATRIC 4mg/sachet granules |
| 722 | cA1y. | MONTELUKAST 4mg/sachet granules |
| 723 | cA1z. | MONTELUKAST 4mg chewable tablets |
| 724 | cA2.. | ZAFIRLUKAST |
| 725 | cA21. | ZAFIRLUKAST 20mg tablets |
| 726 | cA22. | ACCOLATE 20mg tablets |
| 727 | ck1.. | OMALIZUMAB |
| 728 | ck11. | OMALIZUMAB 150mg injection(pdr for recon)+solvent |
| 729 | ck12. | XOLAIR 150mg injection(pdr for recon)+solvent |
| 730 | ck13. | OMALIZUMAB 75mg/0.5mL soln for injection prefilled syringe |
| 731 | ck14. | XOLAIR 75mg/0.5mL solution for injection prefilled syringe |
| 732 | ck15. | OMALIZUMAB 150mg/1mL soln for injection prefilled syringe |
| 733 | ck16. | XOLAIR 150mg/1mL solution for injection prefilled syringe |
| 734 | cl… | PDE4 INHIBITORS |
| 735 | cl1.. | ROFLUMILAST |
| 736 | cl11. | DAXAS 500micrograms tablets |
| 737 | cl1z. | ROFLUMILAST 500micrograms tablets |
| 738 | o323. | SEEBRI BREEZHALER 44micrograms inhalation capsules |

**Supplementary Table 3. Medication groups**

| 1 | Aminophylline |
| --- | --- |
| 2 | Antihistamine |
| 3 | Theophylline |
| 4 | Inhaler Corticosteroids (ICS) |
| 5 | Long-acting Beta Agonists (LABA) |
| 6 | Short-acting Beta Agonists (SABA) |
| 7 | Leukotrienes Antagonists (LTRA) |
| 8 | Long-acting Muscarinic Antagonists (LAMA) |
| 9 | Short-acting Muscarinic Antagonists (SAMA) |
| 10 | Mast Cell Stabilizers (MCS) |
| 11 | Mono-clonal Antibodies (MCA) |
| 12 | Phosphodiesterase Inhibitor (PDEi) |
| 13 | Alpha Adrenoreceptor Agonists + Beta Adrenoceptor Agonists |
| 14 | Alpha Adrenoceptor Agonists + Beta Adrenoceptor Agonists + Phosphodiesterase Inhibitor |
| 15 | Theophylline + Adrenaline |
| 16 | Theophylline + Adrenaline + Phenobarbital |
| 17 | Inhaled Corticosteroids + Long-acting Beta Agonists (ICS + LABA) |
| 18 | Long-acting Beta Agonists + Long-acting Muscarinic Antagonists (LABA + LAMA) |
| 19 | Long-acting Beta Agonists + Short-acting Muscarinic Antagonists (LABA + SAMA) |
| 20 | Mast Cell Stabilizers + Short-acting Beta Agonists (MCS + SABA) |
| 21 | Short-acting Beta Agonists + Short-acting Muscarinic Antagonists (SABA + SAMA) |

**Supplementary Table 4. Read codes for ADHD diagnosis**

| **#** | **Read codes for ADHD diagnosis** | **Description** |
| --- | --- | --- |
| 1 | 6A61. | Attention deficit hyperactivity disorder annual review |
| 2 | 8BPT. | Drug therapy for ADHD (attention deficit hyperactivity disorder) |
| 3 | 8BPT0 | Stimulant drug therapy for ADHD (attention deficit hyperactivity disorder) |
| 4 | 8BPT1 | Non-stimulant drug therapy for ADHD (attention deficit hyperactivity disorder) |
| 5 | 9Ngp. | On drug therapy for ADHD (attention deficit hyperactivity disorder) |
| 6 | 9Ngp0 | On stimulant drug therapy for ADHD (attention deficit hyperactivity disorder) |
| 7 | 9Ngp1 | On non-stimulant drug therapy for ADHD (attention deficit hyperactivity disorder) |
| 8 | 9Ol8. | Attention deficit hyperactivity disorder monitoring invitation first letter |
| 9 | 9Ol9. | Attention deficit hyperactivity disorder monitoring invitation second letter |
| 10 | 9OlA. | Attention deficit hyperactivity disorder monitoring invitation third letter |
| 11 | E2E.. | Childhood hyperkinetic syndrome |
| 12 | E2E0. | Child attention deficit disorder |
| 13 | E2E00 | Attention deficit without hyperactivity |
| 14 | E2E01 | Attention deficit with hyperactivity |
| 15 | E2E0z | Child attention deficit disorder NOS |
| 16 | E2E1. | Hyperkinesis with developmental delay |
| 17 | E2E2. | Hyperkinetic conduct disorder |
| 18 | E2Ey. | Other hyperkinetic manifestation |
| 19 | E2Ez. | Hyperkinetic syndrome NOS |
| 20 | Eu90. | [X]Hyperkinetic disorders |
| 21 | Eu900 | [X]Disturbance of activity and attention |
| 22 | Eu901 | [X]Hyperkinetic conduct disorder |
| 23 | Eu902 | Deficits in attention motor control and perception |
| 24 | Eu90y | [X]Other hyperkinetic disorders |
| 25 | Eu90z | [X]Hyperkinetic disorder, unspecified |
| 26 | Eu9y7 | Attention deficit disorder |

**Supplementary Table 5. Read codes of ADHD medications**

| **#** | **Read codes for ADHD medication** | **Description** |
| --- | --- | --- |
| 1 | db... | WEAK CENTRAL NERVOUS SYSTEM STIMULANTS |
| 2 | db1.. | *PEMOLINE |
| 3 | db11. | *VOLITAL 20mg tablets |
| 4 | db1z. | *PEMOLINE 20mg tablets |
| 5 | db2.. | WEAK CENTRAL NERVOUS SYSTEM STIMULANT + VITAMINS |
| 6 | db21. | *VILLESCON tablets |
| 7 | db22. | *VILLESCON liquid 150mL |
| 8 | dc1.. | DEXAMFETAMINE SULFATE |
| 9 | dc11. | *DEXEDRINE 5mg tablets |
| 10 | dc12. | *DUROPHET 7.5mg m/r capsules |
| 11 | dc13. | *DUROPHET 12.5mg m/r capsules |
| 12 | dc14. | *DUROPHET 20mg m/r capsules |
| 13 | dc1v. | DEXAMFETAMINE SULFATE 1mg/mL oral solution |
| 14 | dc1w. | DEXAMFETAMINE SULFATE 5mg tablets |
| 15 | dc1x. | DEXAMPHETAMINE SULPHATE 7.5mg m/r capsules |
| 16 | dc1y. | DEXAMPHETAMINE SULPHATE 12.5mg m/r capsules |
| 17 | dc1z. | DEXAMPHETAMINE SULPHATE 20mg m/r capsules |
| 18 | dw... | DRUGS USED TO TREAT HYPERACTIVITY DISORDERS |
| 19 | dw1.. | METHYLPHENIDATE |
| 20 | dw11. | METHYLPHENIDATE HYDROCHLORIDE 10mg tablets |
| 21 | dw12. | RITALIN 10mg tablets |
| 22 | dw13. | *EQUASYM 5mg tablets |
| 23 | dw14. | *EQUASYM 20mg tablets |
| 24 | dw15. | *EQUASYM 10mg tablets |
| 25 | dw16. | EQUASYM XL 20mg m/r capsules |
| 26 | dw17. | CONCERTA XL 18mg m/r tablets |
| 27 | dw18. | CONCERTA XL 36mg m/r tablets |
| 28 | dw19. | *TRANQUILYN 5mg tablets |
| 29 | dw1A. | *TRANQUILYN 10mg tablets |
| 30 | dw1B. | *TRANQUILYN 20mg tablets |
| 31 | dw1C. | EQUASYM XL 10mg m/r capsules |
| 32 | dw1D. | EQUASYM XL 30mg m/r capsules |
| 33 | dw1E. | MEDIKINET XL 10mg m/r capsules |
| 34 | dw1F. | MEDIKINET XL 20mg m/r capsules |
| 35 | dw1G. | MEDIKINET XL 30mg m/r capsules |
| 36 | dw1H. | MEDIKINET XL 40mg m/r capsules |
| 37 | dw1I. | CONCERTA XL 27mg m/r tablets |
| 38 | dw1J. | MEDIKINET 5mg tablets |
| 39 | dw1K. | MEDIKINET 10mg tablets |
| **#** | **Read codes for ADHD medication** | **Description** |
| 40 | dw1L. | MEDIKINET 20mg tablets |
| 41 | dw1M. | MEDIKINET XL 5mg m/r capsules |
| 42 | dw1N. | MEDIKINET XL 50mg m/r capsules |
| 43 | dw1O. | MEDIKINET XL 60mg m/r capsules |
| 44 | dw1P. | MATORIDE XL 18mg m/r tablets |
| 45 | dw1Q. | MATORIDE XL 36mg m/r tablets |
| 46 | dw1R. | MATORIDE XL 54mg m/r tablets |
| 47 | dw1S. | XENIDATE XL 18mg m/r tablets |
| 48 | dw1T. | XENIDATE XL 36mg m/r tablets |
| 49 | dw1U. | CONCERTA XL 54mg m/r tablets |
| 50 | dw1n. | METHYLPHENIDATE HYDROCHLORIDE 54mg m/r tablets |
| 51 | dw1o. | METHYLPHENIDATE HYDROCHLORIDE 50mg m/r capsules |
| 52 | dw1p. | METHYLPHENIDATE HYDROCHLORIDE 60mg m/r capsules |
| 53 | dw1q. | METHYLPHENIDATE HYDROCHLORIDE 5mg m/r capsules |
| 54 | dw1r. | METHYLPHENIDATE HYDROCHLORIDE 27mg m/r tablets |
| 55 | dw1s. | METHYLPHENIDATE HYDROCHLORIDE 40mg m/r capsules |
| 56 | dw1t. | METHYLPHENIDATE HYDROCHLORIDE 10mg m/r capsules |
| 57 | dw1u. | METHYLPHENIDATE HYDROCHLORIDE 30mg m/r capsules |
| 58 | dw1v. | METHYLPHENIDATE HYDROCHLORIDE 36mg m/r tablets |
| 59 | dw1w. | METHYLPHENIDATE HYDROCHLORIDE 18mg m/r tablets |
| 60 | dw1x. | METHYLPHENIDATE HYDROCHLORIDE 20mg m/r capsules |
| 61 | dw1y. | METHYLPHENIDATE HYDROCHLORIDE 5mg tablets |
| 62 | dw1z. | METHYLPHENIDATE HYDROCHLORIDE 20mg tablets |
| 63 | dw2.. | ATOMOXETINE |
| 64 | dw21. | STRATTERA 10mg capsules |
| 65 | dw22. | STRATTERA 18mg capsules |
| 66 | dw23. | STRATTERA 25mg capsules |
| 67 | dw24. | STRATTERA 40mg capsules |
| 68 | dw25. | STRATTERA 60mg capsules |
| 69 | dw26. | STRATTERA 80mg capsules |
| 70 | dw27. | STRATTERA 100mg capsules |
| 71 | dw28. | STRATTERA 4mg/mL oral solution |
| 72 | dw2s. | ATOMOXETINE 4mg/mL oral solution |
| 73 | dw2t. | ATOMOXETINE 100mg capsules |
| 74 | dw2u. | ATOMOXETINE 80mg capsules |
| 75 | dw2v. | ATOMOXETINE 60mg capsules |
| 76 | dw2w. | ATOMOXETINE 40mg capsules |
| 77 | dw2x. | ATOMOXETINE 25mg capsules |
| 78 | dw2y. | ATOMOXETINE 18mg capsules |
| 79 | dw2z. | ATOMOXETINE 10mg capsules |
| 80 | dw3.. | LISDEXAMFETAMINE |
| **#** | **Read codes for ADHD medication** | **Description** |
| 81 | dw31. | ELVANSE 30mg capsules |
| 82 | dw32. | ELVANSE 50mg capsules |
| 83 | dw33. | ELVANSE 70mg capsules |
| 84 | dw34. | ELVANSE ADULT 30mg capsules |
| 85 | dw35. | ELVANSE ADULT 50mg capsules |
| 86 | dw36. | ELVANSE ADULT 70mg capsules |
| 87 | dw37. | ELVANSE 20mg capsules |
| 88 | dw38. | ELVANSE 40mg capsules |
| 89 | dw39. | ELVANSE 60mg capsules |
| 90 | dw3u. | LISDEXAMFETAMINE DIMESYLATE 60mg capsules |
| 91 | dw3v. | LISDEXAMFETAMINE DIMESYLATE 40mg capsules |
| 92 | dw3w. | LISDEXAMFETAMINE DIMESYLATE 20mg capsules |
| 93 | dw3x. | LISDEXAMFETAMINE DIMESYLATE 70mg capsules |
| 94 | dw3y. | LISDEXAMFETAMINE DIMESYLATE 50mg capsules |
| 95 | dw3z. | LISDEXAMFETAMINE DIMESYLATE 30mg capsules |
| 96 | dw4.. | GUANFACINE |
| 97 | dw41. | INTUNIV 1mg m/r tablets |
| 98 | dw42. | GUANFACINE 1mg m/r tablets |
| 99 | dw43. | INTUNIV 2mg m/r tablets |
| 100 | dw44. | GUANFACINE 2mg m/r tablets |
| 101 | dw45. | INTUNIV 3mg m/r tablets |
| 102 | dw46. | GUANFACINE 3mg m/r tablets |
| 103 | dw47. | INTUNIV 4mg m/r tablets |
| 104 | dw48. | GUANFACINE 4mg m/r tablets |
| 105 | dz... | OTHER CENTRAL NERVOUS SYSTEM STIMULANTS |
| 106 | dz1.. | MODAFINIL |
| 107 | dz11. | MODAFINIL 100mg tablets |
| 108 | dz12. | PROVIGIL 100mg tablets |
| 109 | dz13. | MODAFINIL 200mg tablets |
| 110 | dz14. | PROVIGIL 200mg tablets |

**Supplementary Table 6. Characteristics and outcomes of cohort participants by presence or absence of maternal asthma**

|  | **No maternal asthma** | | **Maternal asthma*** | | **Total** | | **P-value** |
| --- | --- | --- | --- | --- | --- | --- | --- |
|  | **N=156,841** | | **N=22,183** | | **N=179,024** | |  |
|  | Mean (SD) | | Mean (SD) | | Mean (SD) | |  |
| **Child’s age (years)** | 5.68 (1.11) | | 5.67 (1.11) | | 5.68 (1.11) | | 0.767 |
|  | N | % | N | % | N | % |  |
| **Child's sex** |  |  |  |  |  |  |  |
| Female | 76,868 | 49.5 | 10,778 | 48.9 | 87,646 | 49.4 | 0.238 |
| Male | 79,973 | 50.5 | 11,405 | 51.1 | 91,378 | 50.6 |  |
|  |  |  |  |  |  |  |  |
| **Child's ethnicity** |  |  |  |  |  |  |  |
| White | 146,972 | 95.4 | 21,094 | 95.8 | 168,066 | 95.4 | <0.001 |
| Asian/Asian British | 3,290 | 1.2 | 262 | 0.9 | 3,552 | 1.2 |  |
| Black/ Black British | 752 | 0.3 | 51 | 0.1 | 803 | 0.3 |  |
| Dual heritage | 4,730 | 2.6 | 657 | 2.9 | 5,387 | 2.7 |  |
| Other | 992 | 0.4 | 107 | 0.4 | 1,099 | 0.4 |  |
| Missing | 105 | - | 12 | - | 117 | - |  |
|  |  |  |  |  |  |  |  |
| **Maternal age (years)** |  |  |  |  |  |  |  |
| <25 | 42,871 | 27.6 | 6,431 | 29.5 | 49,302 | 27.8 | <0.001 |
| 25-29 | 46,079 | 29.8 | 6,622 | 30.3 | 52,701 | 29.8 |  |
| 30-34 | 42,033 | 26.5 | 5,603 | 24.5 | 47,636 | 26.3 |  |
| ≥35 | 25,841 | 16.1 | 3,525 | 15.7 | 29,366 | 16.1 |  |
| Missing | 17 | - | 2 | - | 19 | - |  |
|  |  |  |  |  |  |  |  |
| **Maternal smoking status** |  |  |  |  |  |  |  |
| Non-smoker | 65,564 | 41.8 | 9,377 | 42.3 | 74,941 | 41.9 | <0.001 |
| Ex smoker | 2,315 | 1.5 | 627 | 2.8 | 2,942 | 1.6 |  |
| Quit smoking during pregnancy | 5,112 | 3.3 | 1,187 | 5.4 | 6,299 | 3.5 |  |
| Current smoker | 25,296 | 16.1 | 5,161 | 23.3 | 30,457 | 17 |  |
| Missing | 58,554 | 37.3 | 5,831 | 26.3 | 64,385 | 36 |  |
|  |  |  |  |  |  |  |  |
| **Birthweight centile** |  |  |  |  |  |  |  |
| 1-3 | 5,017 | 3 | 816 | 4 | 5,833 | 3.1 | <0.001 |
| 4-10 | 11,382 | 7.1 | 1,767 | 7.9 | 13,149 | 7.3 |  |
| 11-20 | 15,841 | 10.2 | 2,338 | 10.4 | 18,179 | 10.2 |  |
| 21-80 | 93,273 | 60.2 | 13,022 | 59.4 | 106,295 | 60.1 |  |
| 81-90 | 15,247 | 9.7 | 2,035 | 9.4 | 17,282 | 9.6 |  |
| 91-97 | 10,664 | 6.9 | 1,415 | 6.1 | 12,079 | 6.8 |  |
| 98-100 | 4,633 | 2.9 | 641 | 2.8 | 5,274 | 2.9 |  |
| Missing | 784 | - | 149 | - | 933 | - |  |
|  |  |  |  |  |  |  |  |
| **Gestational age (weeks)** |  |  |  |  |  |  |  |
| <28 | 401 | 0.1 | 45 | 0.1 | 446 | 0.1 | <0.001 |
| 28-32 | 1,785 | 1.0 | 243 | 1.0 | 2,028 | 1.0 |  |
| 33-36 | 8,693 | 5.6 | 1,337 | 6.1 | 10,030 | 5.6 |  |
| 37 | 9,130 | 6.1 | 1,465 | 7.0 | 10,595 | 6.2 |  |
| 38 | 18,454 | 12.2 | 2,769 | 13.1 | 21,223 | 12.4 |  |
| 39 | 35,407 | 22.9 | 4,907 | 22.2 | 40,314 | 22.8 |  |
| 40 | 43,477 | 27.7 | 5,924 | 26.7 | 49,401 | 27.6 |  |
| 41 | 32,063 | 20.6 | 4,407 | 20.0 | 36,470 | 20.5 |  |
| ≥42 | 6,821 | 3.8 | 971 | 3.7 | 7,792 | 3.8 |  |
| Missing | 610 | - | 115 | - | 725 | - |  |
|  |  |  |  |  |  |  |  |
| **Mode of delivery** |  |  |  |  |  |  |  |
| Spontaneous vaginal | 66,866 | 42.6 | 9,196 | 41.5 | 76,062 | 42.5 | <0.001 |
| Assisted | 12,106 | 7.7 | 1,714 | 7.7 | 13,820 | 7.7 |  |
| Breech | 430 | 0.3 | 67 | 0.3 | 497 | 0.3 |  |
| Elective CS | 12,430 | 7.9 | 1,943 | 8.8 | 14,373 | 8.0 |  |
| Emergency CS | 16,502 | 10.5 | 2,466 | 11.1 | 18,968 | 10.6 |  |
| Missing | 48,507 | 30.9 | 6,797 | 30.6 | 55,304 | 30.9 |  |
|  |  |  |  |  |  |  |  |
| **Parity** |  |  |  |  |  |  |  |
| 0 | 65,647 | 45.5 | 9,406 | 46.2 | 75,053 | 45.6 | 0.099 |
| 1 | 44,977 | 33.5 | 6,036 | 31.7 | 51,013 | 33.2 |  |
| ≥2 | 27,732 | 21 | 4,212 | 22.1 | 31,944 | 21.2 |  |
| Missing | 18,485 | - | 2,529 | - | 21,014 | - |  |
|  |  |  |  |  |  |  |  |
| **5-minute Apgar score** |  |  |  |  |  |  |  |
| 0-3 | 278 | 0.2 | 40 | 0.2 | 318 | 0.2 | 0.551 |
| 4-6 | 1,495 | 1.1 | 223 | 1.2 | 1,718 | 1.1 |  |
| 7-10 | 147,600 | 98.7 | 20,915 | 98.7 | 168,515 | 98.7 |  |
| Missing | 7,468 | - | 1,005 | - | 8,473 | - |  |
|  |  |  |  |  |  |  |  |
| **WIMD quintile** |  |  |  |  |  |  |  |
| 1 (most deprived) | 41,213 | 24.6 | 6,129 | 25.7 | 47,342 | 24.8 | <0.001 |
| 2 | 34,833 | 23.1 | 4,962 | 22.8 | 39,795 | 23 |  |
| 3 | 30,872 | 20.9 | 4,388 | 21.5 | 35,260 | 21 |  |
| 4 | 24,657 | 16.9 | 3,407 | 16.4 | 28,064 | 16.9 |  |
| 5 (least deprived) | 25,223 | 14.5 | 3,291 | 13.6 | 28,514 | 14.3 |  |
| Missing | 43 | - | 6 | - | 49 | - |  |
|  |  |  |  |  |  |  |  |
| **Number of births** |  |  |  |  |  |  |  |
| 1 (Singletons) | 152,283 | 97.3 | 21,566 | 97.1 | 173,849 | 97.3 | 0.299 |
| ≥2 (Multiples) | 4,558 | 2.7 | 617 | 2.9 | 5,175 | 2.7 |  |
|  |  |  |  |  |  |  |  |
| **Special education need** | 43,754 | 27.9 | 7,201 | 32.5 | 50,955 | 28.5 | <0.001 |
| ASD | 3,103 | 2.0 | 565 | 2.5 | 3,668 | 2 | <0.001 |
| Communication problems | 17,485 | 11.1 | 2,806 | 12.6 | 20,291 | 11.3 | <0.001 |
| Behavioural, emotional and social difficulties | 10,216 | 6.5 | 1,899 | 8.6 | 12,115 | 6.8 | <0.001 |
| Learning difficulties | 24,135 | 15.4 | 3,953 | 17.8 | 28,088 | 15.7 | <0.001 |
| Physical and medical difficulties | 2,823 | 1.8 | 521 | 2.3 | 3,344 | 1.9 | <0.001 |
| Sensory impairment | 1,676 | 1.1 | 301 | 1.4 | 1,977 | 1.1 | <0.001 |
|  |  |  |  |  |  |  |  |
| **ADHD** | 2,688 | 1.7 | 599 | 2.7 | 3287 | 1.8 | <0.001 |

*record of asthma diagnosis and/or record of asthma medication

SD standard deviation; N number; CS Caesarean section; WIMD Welsh Index of Multiple Deprivation; ASD autistic spectrum disorder; ADHD attention deficit hyperactivity disorder

**Supplementary Table 7. Associations between treated and untreated maternal asthma and childhood outcomes**

|  | **Untreated maternal asthma** | | **Maternal asthma-medication** | | **Treated maternal asthma** | |
| --- | --- | --- | --- | --- | --- | --- |
|  | N=142,691 | | N=153,580 | | N=148,909 | |
|  | OR (95% CI) | P-value | OR (95% CI) | P-value | OR (95% CI) | P-value |
|  |  |  |  |  |  |  |
| Special education need | 1.14 (1.07-1.23) | <0.001 | 1.22 (1.17-1.26) | <0.001 | 1.27 (1.22-1.33) | <0.001 |
| ASD | 1.16 (0.92-1.45) | 0.200 | 1.19 (1.05-1.36) | 0.007 | 1.19 (1.02-1.39) | 0.024 |
| Communication problems | 1.11 (1.00-1.23) | 0.054 | 1.11 (1.04-1.17) | <0.001 | 1.17 (1.09-1.25) | <0.001 |
| Behavioural, emotional and social difficulties | 1.27 (1.12-1.44) | <0.001 | 1.27 (1.18-1.36) | <0.001 | 1.28 (1.17-1.39) | <0.001 |
| Learning difficulties | 1.05 (0.96-1.15) | 0.271 | 1.18 (1.13-1.24) | <0.001 | 1.23 (1.17-1.31) | <0.001 |
| Physical and medical difficulties | 1.27 (1.01-1.59) | 0.044 | 1.25 (1.09-1.42) | <0.001 | 1.34 (1.15-1.56) | <0.001 |
| Sensory impairment | 1.55 (1.17-2.05) | 0.002 | 1.21 (1.02-1.44) | 0.029 | 1.37 (1.13-1.67) | 0.001 |
|  |  |  |  |  |  |  |
|  | N= 128,208 | | N= 138,047 | | N= 133,842 | |
|  | HR (95% CI) | P-value | HR (95% CI) | P-value | HR (95% CI) | P-value |
| ADHD | 1.70 (1.42-2.05) | <0.001 | 1.37 (1.22-1.54) | <0.001 | 1.48 (1.30-1.69) | <0.001 |
|  |  |  |  |  |  |  |

N number; CI confidence interval; ASD autistic spectrum disorder; ADHD attention deficit hyperactivity disorder; HR hazard ratio

OR odds ratio; SABA short-acting beta agonist; LABA long-acting beta agonist; ICS inhaled corticosteroid; LTRA leukotriene receptor antagonists

**Supplementary Table 8. Associations between different combinations of classes of asthma medications and childhood outcomes**

|  | **SABA + ICS** | | **SABA + ICS + ICS plus LABA^a^** | | **SABA + ICS + LABA** | |
| --- | --- | --- | --- | --- | --- | --- |
|  | N=142,663 | | N=138,640 | | N=138,535 | |
|  | OR (95% CI) | P-value | OR (95% CI) | P-value | OR (95% CI) | P-value |
| Special education needs | 1.28 (1.20 - 1.37) | <0.001 | 1.40 (1.11 - 1.76) | 0.004 | 1.54 (1.17 - 2.03) | 0.002 |
| ASD | 1.34 (1.07 - 1.67) | 0.010 | 1.02 (0.51 - 2.04) | 0.954 | 0.80 (0.29 - 2.16) | 0.654 |
| Communication problems | 1.18 (1.06 - 1.31) | 0.002 | 1.38 (0.98 - 1.93) | 0.067 | 1.20 (0.78 - 1.84) | 0.400 |
| Behavioural, emotional and social difficulties | 1.27 (1.12 - 1.44) | <0.001 | 1.38 (0.91 - 2.08) | 0.130 | 1.44 (0.87 - 2.36) | 0.155 |
| Learning difficulties | 1.25 (1.15 - 1.36) | <0.001 | 1.50 (1.14 -1.99) | 0.004 | 1.28 (0.91 - 1.81) | 0.162 |
| Physical and medical difficulties | 1.25 (0.98 - 1.58) | 0.068 | 1.12 (0.49 - 2.59) | 0.786 | 1.99 (1.01 - 3.93) | 0.048 |
| Sensory impairment | 1.29 (0.95 - 1.75) | 0.104 | 1.25 (0.45 - 3.47) | 0.668 | 0.54 (0.07 - 3.83) | 0.534 |
|  |  |  |  |  |  |  |
|  | N=128,209 | | N= 124,613 | | N= 124,512 | |
|  | HR (95% CI) | P-value | HR (95% CI) | P-value | HR (95% CI) | P-value |
| ADHD | 1.48 (1.22 - 1.81) | <0.001 | 1.35 (0.67 - 2.71) | 0.395 | 2.07 (1.07 - 3.99) | 0.030 |
|  |  |  |  |  |  |  |
|  | **SABA + ICS + LTRA** | | **SABA + ICS + LABA + ICS plus LABA** | |  |  |
|  | N=138,340 | | N=138,349 | |  |  |
|  | OR (95% CI) | P-value | OR (95% CI) | P-value |  |  |
| Special education needs | 1.12 (0.51 - 2.48) | 0.770 | 1.29 (0.66 - 2.52) | 0.448 |  |  |
| ASD | 2.03 (0.27 - 15.55) | 0.494 | 0.29 (0.04 - 2.13) | 0.221 |  |  |
| Communication problems | 0.60 (0.15 - 2.35) | 0.462 | 1.29 (0.53 - 3.12) | 0.572 |  |  |
| Behavioural, emotional and social difficulties | 3.03 (1.13 - 8.11) | 0.027 | 0.80 (0.17 - 3.74) | 0.781 |  |  |
| Learning difficulties | 1.17 (0.42 - 3.27) | 0.762 | 1.54 (0.74 - 3.22) | 0.248 |  |  |
| Physical and medical difficulties | 6.07 (1.46 - 25.26) | 0.013 | - | - |  |  |
| Sensory impairment | - | - | - | - |  |  |
|  |  |  |  |  |  |  |
|  | N=124,341 | | N=124,347 | |  |  |
|  | HR (95% CI) | P-value | HR (95% CI) | P-value |  |  |
| ADHD | 3.06 (0.43 - 21.75) | 0.264 | 4.45 (1.43 - 13.84) | 0.010 |  |  |
|  |  |  |  |  |  |  |

N number; CI confidence interval; ASD autistic spectrum disorder; ADHD attention deficit hyperactivity disorder; HR hazard ratio; OR odds ratio; SABA short-acting beta agonist; ICS inhaled corticosteroid; LABA long-acting beta agonist; LTRA leukotrienes antagonists

^a^ “ICS plus LABA” represents the fixed-dose combination inhaler that contains both active ingredients

1. Pink lines represent the confounding effect, whereas green lines represent the mediating effect. [↑](#footnote-ref-1)
